# Hydroxypropylmethylcellulose as a film and hydrogel carrier for ACP nanoprecursors to deliver biomimetic mineralization

**DOI:** 10.1186/s12951-021-01133-7

**Published:** 2021-11-22

**Authors:** Zhe Wang, Zihuai Zhou, Jiayan Fan, Leiqing Zhang, Zhixin Zhang, Zhifang Wu, Ying Shi, Haiyan Zheng, Zhengyi Zhang, Ruikang Tang, Baiping Fu

**Affiliations:** 1 Stomatology Hospital, School of Stomatology, Zhejiang University School of Medicine, Zhejiang Provincial Engineering Research Center for Oral Biomaterials and Devices, Zhejiang Provincial Clinical Research Center for Oral Diseases, Key Laboratory of Oral Biomedical Research of Zhejiang Province, Cancer Center of Zhejiang University, Hangzhou, 310006 China; 2 Department of Chemistry, Zhejiang University, Hangzhou, 310027 Zhejiang China

**Keywords:** Dental caries, Hydroxypropylmethylcellulose, Film, Biomimetic mineralization, Dentin, Collagen

## Abstract

Demineralization of hard tooth tissues leads to dental caries, which cause health problems and economic burdens throughout the world. A biomimetic mineralization strategy is expected to reverse early dental caries. Commercially available anti-carious mineralizing products lead to inconclusive clinical results because they cannot continuously replenish the required calcium and phosphate resources. Herein, we prepared a mineralizing film consisting of hydroxypropylmethylcellulose (HPMC) and polyaspartic acid-stabilized amorphous calcium phosphate (PAsp-ACP) nanoparticles. HPMC which contains multiple hydroxyl groups is a film-forming material that can be desiccated to form a dry film. In a moist environment, this film gradually changes into a gel. HPMC was used as the carrier of PAsp-ACP nanoparticles to deliver biomimetic mineralization. Our results indicated that the hydroxyl and methoxyl groups of HPMC could assist the stability of PAsp-ACP nanoparticles and maintain their biomimetic mineralization activity. The results further demonstrated that the bioinspired mineralizing film induced the early mineralization of demineralized dentin after 24 h with increasing mineralization of the whole demineralized dentin (3–4 µm) after 72–96 h. Furthermore, these results were achieved without any cytotoxicity or mucosa irritation. Therefore, this mineralizing film shows promise for use in preventive dentistry due to its efficient mineralization capability.

**Supplementary Information:**

The online version contains supplementary material available at 10.1186/s12951-021-01133-7.

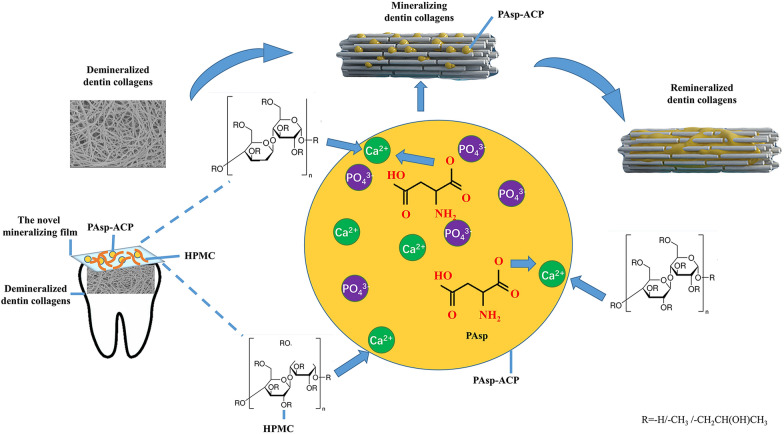

## Background

Dental caries is one of the most prevalent and consequential diseases in the world [1]. The latest data on the global burden of diseases (March 2020) show that over 3.5 billion people suffer from dental caries. However, this disease remains a neglected global health issue [2]. Additionally, it is a predictably increasing social and economic burden [3]. Therefore, we should take measures to prevent dental caries.

Biomimetic design is a promising strategy for generating functional materials that mimic biological processes [4–7]. Recently, scientists have adopted biomimetic mineralization as a minimally invasive method for treating initial caries [8]. Most detectable caries demineralized not only enamel but also dentin. Dentin consists of a mineralized collagen matrix. Biomimetic mineralization is an organics-mediated process involving the formation of heterogeneous crystal nuclei [9]. Most current biomimetic mineralization studies are based on the polymer-induced liquid-precursor (PILP) theory [10]. Polymers can be used as non-collagen proteins (NCPs) analogs to promote the transformation of precursors (amorphous calcium phosphate, ACP) into hydroxyapatite (HAp) during biomimetic mineralization [11]. ACP nanoparticles are believed to be crucial precursors in biomimetic mineralization [12]. However, in aqueous solutions, ACP nanoparticles are easily converted into crystalline phases [13]. Only when ACP nanoparticles are kept dry or stabilized by polyelectrolytes, such as polyaspartic acid (PAsp) or polyacrylic acid (PAA), can they maintain their mineralization activity. In addition, some polymers, such as poly (amide amine) (PAMAM) and polyethylene glycol hydrogels have also been applied as NCPs analogs to stabilize the ACP nanoparticles [14, 15]. Based on the PILP theory, contemporary, commercially available anti-carious materials have been developed to deliver mimetic mineralization in liquids (mouth rinses) and pastes (toothpastes) [16]. However, their dentin remineralization efficacy is not as good as expected because they do not continuously release calcium and phosphate for biomimetic mineralization [17]. Mesoporous silica has been used as a carrier to deliver ACP nanoparticles [18]. Our previous studies demonstrated that acidic, self-etching adhesives can be loaded with PAA-ACP or PAsp-Si-ACP nanoparticles to implement the biomimetic mineralization of demineralized dentin [19, 20].

**Fig. 1:**
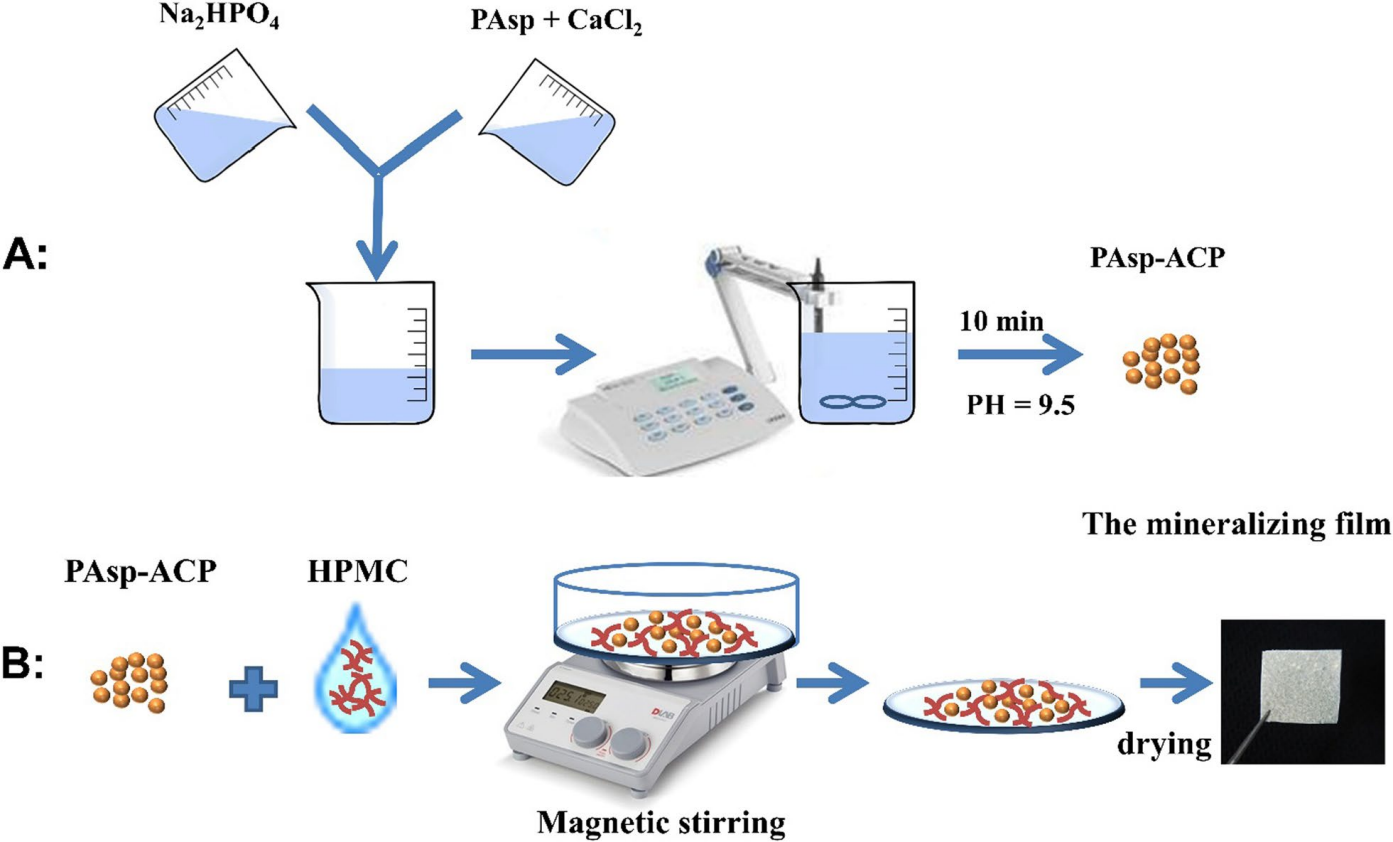
Schematic diagram showing the preparation of the mineralizing film. **A** Preparation of the PAsp-ACP nanoparticles. **B** Preparation of the mineralizing film

Hydroxypropylmethylcellulose (HPMC) is a nontoxic polymer that contains a large number of hydroxyl, methyl and methoxy anion groups which share identical sequences with NCPs [21, 22]. HPMC has been widely used as a drug carrier in pharmaceutical applications because it can be used as a film-forming material [23]. Once a dry HPMC film makes contact with water, its polymer chains relax, its volume expands as it converts into a gel, and the loaded particles can be released [24]. To date, the method by which HPMC can be used as a carrier for ACP nanoparticles to deliver biomimetic mineralization has not yet been investigated. Therefore, in this work, we proposed a film-assisted biomimetic mineralization strategy. We demonstrated the mineralizing film could deliver the biomimetic mineralization without any cytotoxicity or mucosal irritation. Early mineralization of demineralized dentin occurred in the first 24 h, and heavy mineralization of the whole demineralized dentin (3–4 µm) was achieved in 72–96 h. The cryogenic transmission electron microscopy (cryo-TEM) images indicate that when the mineralizing film is exposed to artificial saliva (AS) at 37 °C, it can release ACP nanoparticles early and maintain the bioactivity of these nanoparticles for 6 h in an amorphous phase, these nanoparticles gradually transformed into HAp after 8 h. AS consisting of 1.5 mM CaCl_2_, 0.9 mM KH_2_PO_4_, 130 mM KCl, 1.0 mM NaN_3_ and 20 mM HEPES, pH = 7.0 was prepared according to our previous studies [19, 20]. HPMC was the carrier of the ACP nanoparticles that could enable biomimetic mineralization via the hydroxyl and methoxyl groups of the HPMC synergistically with polyelectrolytes. Hence, the mineralizing film might be a promising mineralizing strategy due to its efficient mineralization capability. A simplified diagram for the preparation of the mineralizing film is shown in Fig. 1.

## Materials and methods

### Synthesis and characterization of ACP and PAsp-stabilized ACP (PAsp-ACP) nanoparticles

ACP and PAsp-ACP nanoparticles were synthesized by mixing equal volumes (25 mL) of 6 mM Na_2_HPO_4_ and 10 mM CaCl_2_ without and with 480 μg mL^−1^ PAsp respectively, and these mixtures were stirred for 10 min at room temperature. During synthesis, the pH of the solution was gradually adjusted to 9.5 ± 0.1 by titrating 5 mol NaOH. The precipitates (ACP/PAsp-ACP nanoparticles) were collected by centrifugation (18,000 rpm, 5 min, F0650, Beckman Allegra64R, USA), and subsequently washed three times with absolute ethanol. Subsequently, the ACP/PAsp-ACP nanoparticles were dried in a vacuum drying oven at 30 °C overnight before they were analyzed by scanning electron microscopy (SEM, HITACHI, SU8010, Tokyo, Japan), transmission electron microscopy (TEM, JEM-1230, JEOL, Tokyo, Japan), powder X-ray diffraction (XRD, Rigaku D/MAX-2550 pc, Japan) and Fourier transform infrared (FTIR) spectroscopy (Spectrum 400, Perkin-Elmer, USA).

### Preparation and characterization of the mineralizing film

The mineralizing film was prepared as follows: HPMC (0.24 g) powder was immersed in 1.5 mL of 70% alcohol for 12 h in order to obtain a HPMC gel. Subsequently, 0.24 g of PAsp-ACP nanoparticles and 2 mL of 70% alcohol were added to HPMC gel, and then the mixture was magnetically stirred at room temperature for 5 min. Afterward, a 2 mm thick mixture was prepared on a glass slide and dried under infrared light for 2 h to form a 40 mm × 15 mm mineralizing film with a thickness of 0.2 mm. Finally, the film was cut into small 5 mm × 5 mm pieces and stored in a desiccator. The mineralizing films were analyzed by SEM, TEM, FTIR and XRD. The pure HPMC film was also prepared with the procedure described above and analyzed by FTIR and XRD.

### ICP-AES measurements

A large piece of the aforementioned mineralizing film was stored in artificial saliva for 24 h. One hundred microliters of the supernatant was retrieved at each time point (4, 8, 12, 16, 20, 24 h) and diluted to 100 mL with deionized water. Afterward, the concentrations of Ca and P were measured by inductively coupled plasma-atomic emission spectroscopy (ICP-AES, Thermo iCAP-6300, USA). These measurements were performed in triplicate.

### Phase transformation of mineralizing film in artificial saliva—FTIR analysis

A large piece of mineralizing film was immersed in 60 mL of artificial saliva in a 100% humidity chamber at 37 °C for 48 h. One milliliter of the mixture was retrieved at each time point (6, 8, 10, 12, 24, 48 h). The solid materials from the mixture were collected by centrifugation, washed three times with absolute ethanol, and dried overnight in a vacuum oven at 30 °C before being analyzed by FTIR. A piece of the pure HPMC film was also analyzed by FTIR. The splitting function (SF) proposed by Posner was used to determine the phase transformation of mineralizing film in artificial saliva [25].

### Phase transformation of the mineralizing film in artificial saliva—cryo-TEM analysis

The phase transformation of the PAsp-ACP nanoparticles in HPMC when exposed to artificial saliva was analyzed at each time point (0, 6, 8, 12 and 24 h) by cryo-TEM (Talos F200C, FEI, USA). The preparation of mineralizing film samples for cryo-TEM are described in the electronic Supporting Information (ESI)†.

### Biocompatibility test—in vitro cytotoxicity test

Cell isolation and culture of L929 and human gingival fibroblasts are described in the ESI†. The cytotoxicity of the mineralizing film was assessed by a Cell Counting Kit-8 (CCK-8) assay (Beyotime, Shanghai, China). Different concentrations of the mineralizing films (0.1, 0.2, 0.3, 0.5, 1, 2, 4, 8 mg mL^−1^) were incubated at 37 °C in Duibecco’s modified Eagle’s medium (DMEM, Gibco, USA) for 24 h to obtain leaching solutions, in which 10% fetal bovine serum (FBS, Gibco, USA) was added to the L929 cells and 15% FBS was added to the human gingival fibroblasts. Afterward, L929 cells and human gingival fibroblasts were planted into 96-well plates at 10,000 cells per well. They were incubated for 24 h to allow cell attachment. Then, the attached cells were incubated with 5% of CO_2_ and 95% relative humidity in each well with the different leach solutions for 1, 3 and 5 days at 37 °C. Afterward, 10 µL CCK-8 was added to each well, and the attached cells were incubated for another 2–3 h. The measurements were performed six times at a wavelength of 450 nm using a microplate reader (Bio Tek Eon, USA).

### Biocompatibility test—in vivo oral mucosa irritation tests

The protocol design was based on the Stimulation and Skin Allergy Test, Appendix B.3 Oral Mucosal Stimulation Test (ISO 10993-10, 2010) [26]. Six male golden hamsters weighing 110–130 g were purchased from Beijing Weitong Lihua [animal production license: SCXK (Beijing) 2016-0011]. After acclimatization for 1 week, these golden hamsters were intraperitoneally injected with 30 g L^−1^sodium pentobarbital (0.002mLg^−1^) on the 8th day. After 0.2 g mineralizing film was immersed in 1 mL of 0.9% NaCl and 1 mL of cottonseed oil for 24 h, a cotton ball with a diameter of approximately 0.40–0.45 cm was soaked with 50 μL of polar (0.9% NaCl) and nonpolar (cottonseed oil) extracts of the mineralizing film. These cottons were subsequently placed in the left cheek pouches of the golden hamster, the right cheek pouches were serving as control. The oral mucosal irritation test was performed for 10 min and repeated four times with 1 h intervals. Additionally, macro- and microscopic histological evaluations of the irritated buccal mucosa were performed. The preparation of buccal mucosa samples for microscopic histological evaluation and its grading system (Additional file 1: Table S1 and S2)  are described in the ESI†.

### Biomimetic mineralization of demineralized dentin—in vitro experiments

The preparation of demineralized dentin disks and the samples for TEM are described in the ESI†. The demineralized dentin disks (N = 36) were randomly divided into three groups (n = 12): the demineralized dentin disk samples were not covered with anything serving as control. The demineralized dentin disk samples were covered either with pure HPMC films or with mineralizing films. Each sample was placed in 1 mL of artificial saliva in a well of 48-well plates, and incubated at 37 °C and 100% humidity. The HPMC films, mineralizing films and artificial saliva were changed daily. Three samples were retrieved after incubation for 1, 2, 3, and 4 days in preparation for ultrathin sections. These sections were analyzed by TEM with selected area electron diffraction (SAED), high resolution TEM (HRTEM) and elemental mapping. The micromechanical properties of the intact, de- and remineralized dentin (4 days) under moist conditions were determined by a Nanoindenter G200 (Agilent, USA) with a Berkovich diamond indenter. The preparations of the samples for indentation test are described in the ESI†. The measurement depth was set at 2500 nm for a peak hold time of 10 s and the Poisson ratio was set at 0.28. Five points were randomly chosen on each dentin disk surface.

### Biomimetic mineralization of demineralized dentin—in vivo experiments

Six healthy, male, 12 weeks old New Zealand rabbits [certificate number: SCXK (Zhe) 2017-0004] that weighing 2.1–2.5 kg were purchased (Hangzhou Yuhang Kelian Rabbit Industry Professional Cooperative, Hangzhou, China) for this study. Demineralized dentin was prepared as follows: Six rabbits were anesthetized with 10% chloral hydrate (0.4 mg kg^−1^, Dopalen, Ceva Sante Animale, Libourne, France), and the labial enamel surfaces of the rabbits’ maxillary and mandibular central incisors were completely removed to expose the dentin surfaces. Afterward, the exposed dentin surfaces were etched with 37% phosphoric acid gel for 15 s and thoroughly rinsed with water. The maxillary central incisors were used for biomimetic mineralization, and the mandibular central incisors served as a blank control. (1) Control group: demineralized dentin surfaces of the mandibular central incisors were directly exposed to the oral cavity without being covered by any mineralized films. (2) Experimental group: transparent custom trays were prepared and the details are in the ESI†. The demineralized dentin surfaces of the maxillary central incisors were covered with mineralizing films and subsequently the central incisors were protected with a transparent splint, 8 h a day for 7 consecutive days. Finally, all rabbits were sacrificed and central incisors were obtained. The incisor samples were divided into ultrathin sections (details are in the ESI†, and examined by TEM with SAED.

## Results and discussion

### Characterization of the ACP and PAsp-ACP nanoparticles

**Fig. 2:**
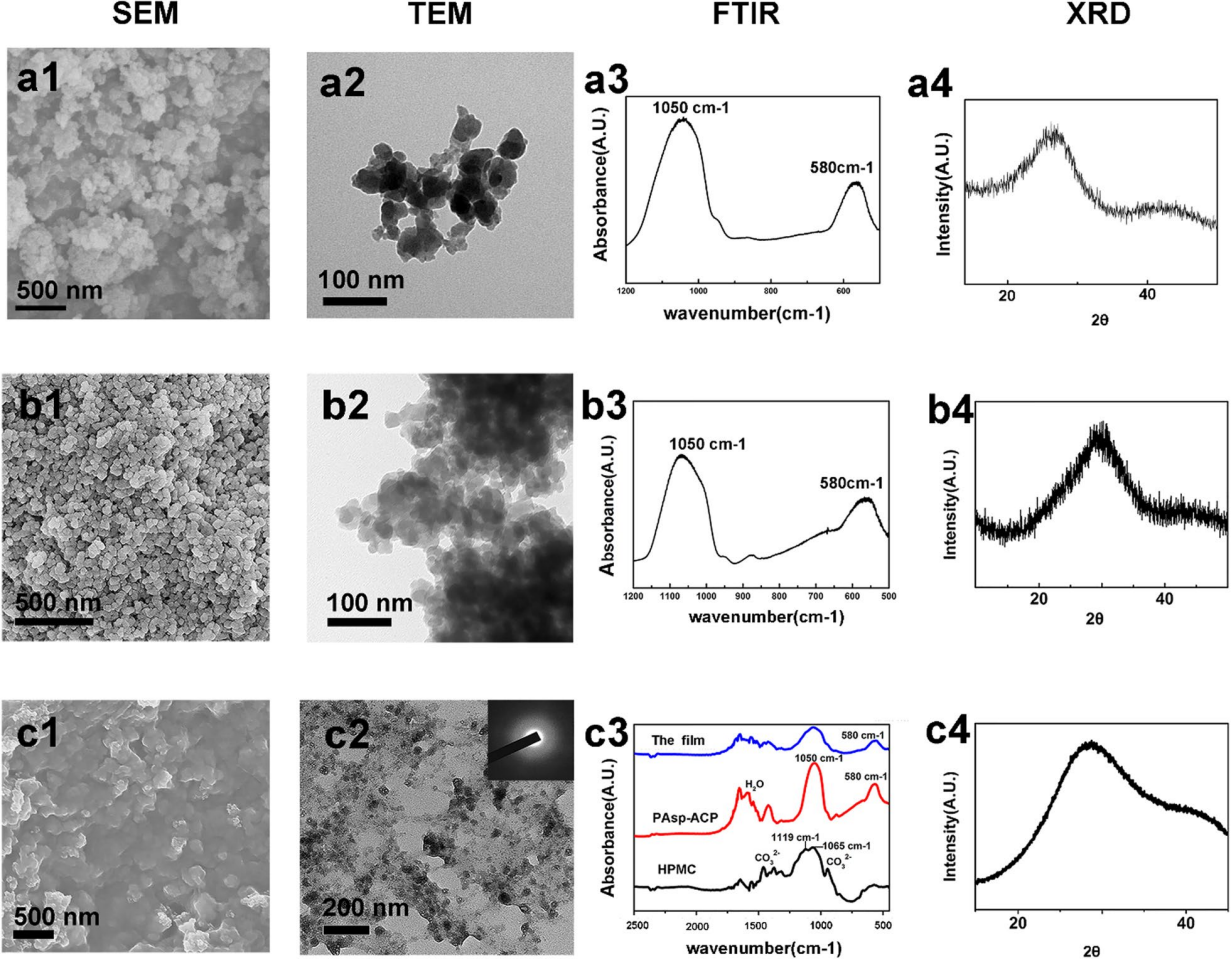
Characterization of ACP (**a1**–**a4**), PAsp-ACP nanoparticles (**b1**–**b4**) and the mineralizing film (**c1**–**c4**). **a1**, **b1** SEM images of ACP and PAsp nanoparticles show the particles were spherical. **a2**, **b2** TEM images of ACP and PAsp nanoparticles show the diameter of an individual nanoparticle is approximately 30–80 nm. **a3**, **b3** FTIR spectra of ACP and PAsp nanoparticles shows characteristic amorphous peaks at 1050 cm^−1^ and 580 cm^−1^. **a4**, **b4** XRD patterns of the ACP and PAsp-ACP nanoparticles show the broad peak, the peak in **a4** is a small left offset compared to that of **b4** and **c4**. **c1** SEM image shows that the PAsp-ACP nanoparticles are homogeneously distributed in the mineralizing film. **c2** TEM image shows that the spherical PAsp-ACP nanoparticles are dispersed in HPMC, and the SAED pattern (inset panel) indicates that the PAsp-ACP nanoparticles are amorphous. **c3** FTIR spectra shows that HPMC exhibits a typical C–O peak at 1065 cm^−1^, C–O–C peak at 1119 cm^−1^, and CO_3_^2−^ peaks at 872 cm^−1^ and 1420 cm^−1^ (black line). The PAsp-ACP nanoparticles exhibit characteristic PO_4_^3−^ absorption peaks at 1050 cm^−1^ and 580 cm^−1^ as well as absorption peaks attributed to bound water at 1300 cm^−1^ and 1750 cm^−1^ (red line). The mineralizing film exhibits characteristic peaks of both HPMC and the ACP nanoparticles (blue line). **c4** The XRD pattern of the mineralizing film displays a broad peak at 2θ = 30°

PAsp-ACP nanoparticles were synthesized and incorporated into HPMC as a dry mineralizing film**.** SEM and TEM micrographs show that ACP and PAsp-ACP nanoparticles are spherical (Fig. 2a1 and b1). The diameter of an individual nanoparticle is approximately 30–80 nm (Fig. 2a2 and b2). As has been reported, the effect of mineralization is related to the size of ACP nanoparticles, ACP nanoparticles with a size of 50–80 nm more easily enter collagens for mineralization [13]. FTIR shows characteristic ACP and PAsp-ACP peaks at 1050 cm^−1^ and 580 cm^−1^ (Fig. 2a3, b3). The X-ray diffraction (XRD) patterns of both the ACP and PAsp-ACP nanoparticles exhibit a broad peak (Fig. 2a4, b4). The broad peak at 2θ = 30° of the PAsp-ACP nanoparticles is slightly offset from that of ACP (Fig. 2a4, b4). This might be due to the change in structure after the addition of PAsp [27, 28]. Both the FTIR spectrum and XRD pattern of the PAsp-ACP nanoparticles indicate that they are amorphous. According to previous studies, the ACP nanoparticles are unstable in solution and easily transform into HAp [12, 28]. As such, the clinical application of the ACP nanoparticles are is limited. PAsp is an NCPs analog that plays an important role in stabilizing ACP, allowing it to enter collagen fibrils for mineralization [28]. Under dry conditions, PAsp-stabilized ACP nanoparticles can easily be kept in an amorphous phase [12, 29].

### Characterization of the mineralizing film

The mineralizing film is very stable under dry conditions and shows promise for clinical application. The SEM images show that the PAsp-ACP nanoparticles are uniformly distributed in the mineralizing film (Fig. 2c1). The TEM images demonstrate that the PAsp-ACP nanoparticles in the mineralizing film have diameters of 30–80 nm and are amorphous (SAED inset, Fig. 2c2). A characteristic FTIR peak is observed at 580 cm^−1^ and is not split, indicating that the PAsp-ACP nanoparticles are amorphous in the mineralizing film (Fig. 2c3, blue line). The absorption peaks between 1300 and 1750 cm^−1^ are attributed to tightly bound water (10–20%) in the PAsp-ACP nanoparticles (Fig. 2c3, red line) [29]. The characteristic HPMC peaks are mainly those observed at 1065 cm^−1^, which are attributed to C–O–C asymmetric stretching vibrations, and 1119 cm^−1^, attributed to C–O stretching vibrations [30]. The absorption peaks at 872 cm^−1^ and 1420 cm^−1^ correspond to the characteristic peaks of CO_3_^2−^ (Fig. 2c3, black line). The XRD pattern exhibits a broad peak at 2θ = 30°, indicating that the PAsp-ACP nanoparticles are amorphous (Fig. 2c4) [27, 28]. Thus, HPMC can be used as a carrier to deliver ACP precursors and promote biomimetic mineralization; HPMC can not only maintain PAsp-ACP precursor bioactivity under dry conditions but can also be easily prepared and applied due to its interchangeable properties (from solid to gel and from gel to solid).

### ICP-AES measurements of the mineralizing film

**Fig. 3:**
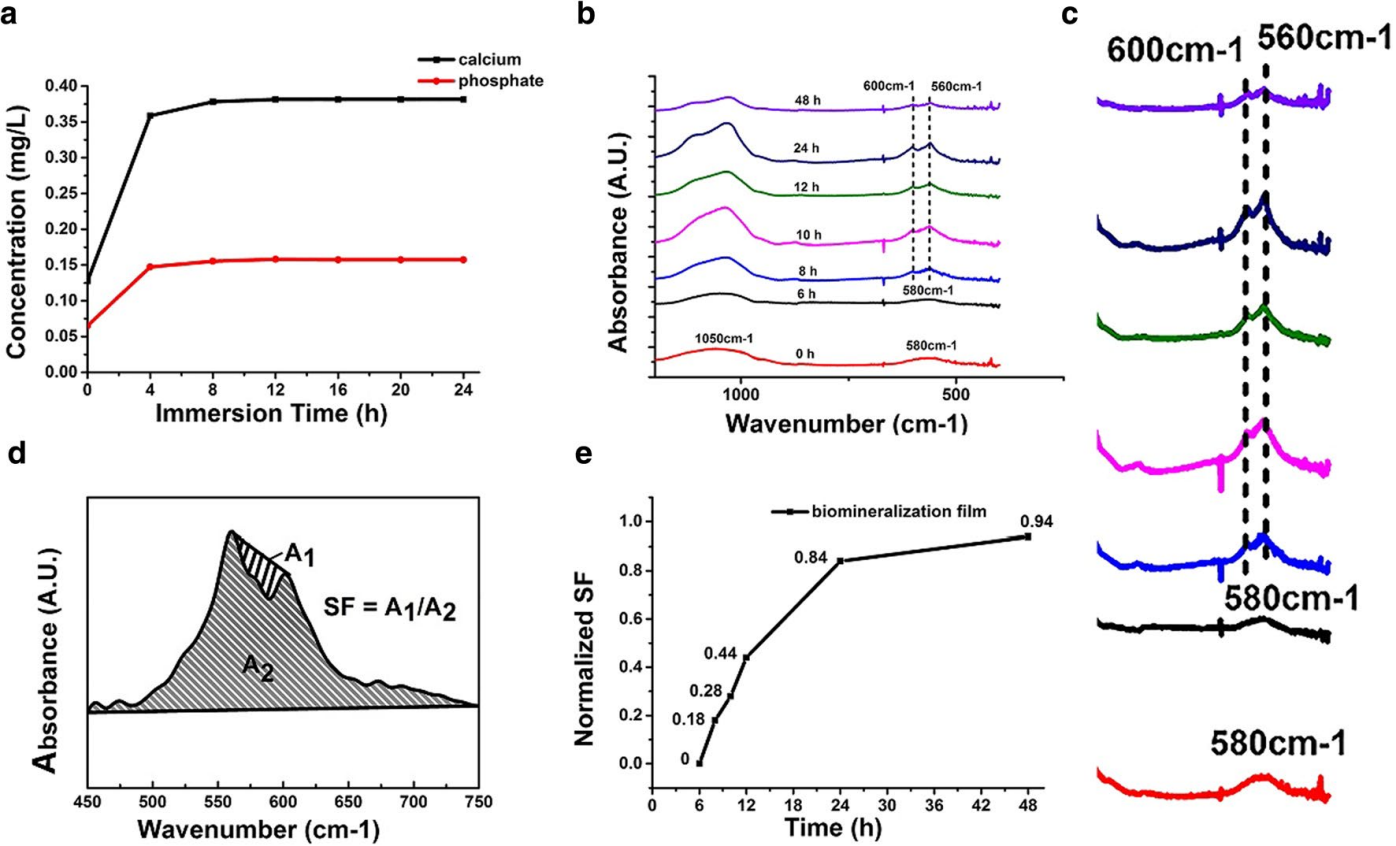
Ca and P release and phase transformations. **a** Release kinetics for calcium (black line) and phosphate (red line) ions from the mineralizing film over 24 h. **b** FTIR spectra of the mineralizing film in AS show a peak at 580 cm^−1^ within 6 h and two peaks at 560 cm^−1^ and 600 cm^−1^ over 8–48 h. **c** Magnified images of the spectral curve ranging from 400 to 750 cm^−1^ of **b**. **d** SF scheme. A1/A2 = 0 (noncrystallization) to 1 (complete crystallization). **e** Kinetics of the ACP to HAp phase transformation

Dentin mineralization is based on calcium and phosphate resources [31, 32]. Figure 3a shows that the amount of Ca and P increased quickly within 4 h after the mineralizing film was incubated in artificial saliva at 37 °C. Between 4 and 24 h, Ca and P were steadily released (Fig. 3a). The release from the mineralizing film started with a burst release at initial stage of 0–4 h and changed to sustained release from 4 to 24 h (Fig. 3a). The two stages of nanoparticles release are consistent with those described in the previous publications [33–36]. Biologically, crystalline apatite formation is initiated with the heterogeneous nucleation of inorganic calcium phosphate on an organic extracellular matrix [32]. The concentrations of free Ca ions and ACP are the main factors for HAp nucleation [37]. The ICP-AES results confirm that the mineralizing film can release ACP nanoparticles. The Ca/P-releasing capability of the mineralizing film suggests that dentin remineralization is possible.

### Phase transformations of the mineralizing film

Once the dry mineralizing films intake artificial saliva, they gradually form sticky gels. In addition, the ACP nanoparticles are released into the artificial saliva and gradually transform into HAp. The phase transformation of ACP nanoparticles in the mineralizing film occurs after 8 h incubation in artificial saliva (Fig. 3b). The nanoparticles are mostly transformed into HAp after 48 h, as demonstrated by the peak at 580 cm^−1^ splitting into two peaks at 560 cm^−1^ and 600 cm^−1^ (Fig. 3b) Fig. 3c indicates that the magnified FTIR images of the spectral curve of panel b ranging from 400 cm to 750 cm^−1^, shows a peak at 580 cm^−1^ within 6 h and two peaks at 560 cm^−1^ and 600 cm^−1^ over 8–48 h (Fig. 3c), and the value of the splitting function (SF) is close to 1, notably, SF evaluates the degree of ACP nanoparticle crystallization, with 0 representing no crystallization and 1 representing complete crystallization (Fig. 3d, e) [25]. Additional file 1: Fig. S1 indicates that in AS, the mineralizing film dynamically changed from a dry film to a gel at 4 h. The peripheral film started to be gel at 1 h and whole film became gel at 4 h. This is supportive of a burst release in the initial stage. The interchanges of gel-to-sol phase transition of HPMC along with release of Ca/P ions in microscopic level led to the inhomogeneous distribution of HMPC gel over 6–24 h. This result is consistent with sustained release of Ca/P ions due to electrostatic attraction between calcium ions and polyhydroxyl of the HPMC. The hydroxyl and carbonyl groups of HPMC can attract calcium ions and delay the crystallization of ACP. This delayed crystallization might slow the release of calcium ions from HPMC gels since pure PAsp-ACP nanoparticles in artificial saliva start to transform into HAp within 2–3 h (Additional file 1: Fig. S2). Moreover, ACP nanoparticles are liquid-like, and HPMC can provide hydrophobic microdomains for hydrophilic particles to facilitate drug release [38, 39]. These results indicate that HPMC is not only a good carrier for ACP precursors but can also stabilize ACP nanoparticles. Therefore, HPMC and PAsp might have a synergistic stabilizing effect on ACP nanoparticles. The mineralizing film allows the ACP nanoparticles enough time to enter and mineralize dentin collagen fibrils.

**Fig. 4:**
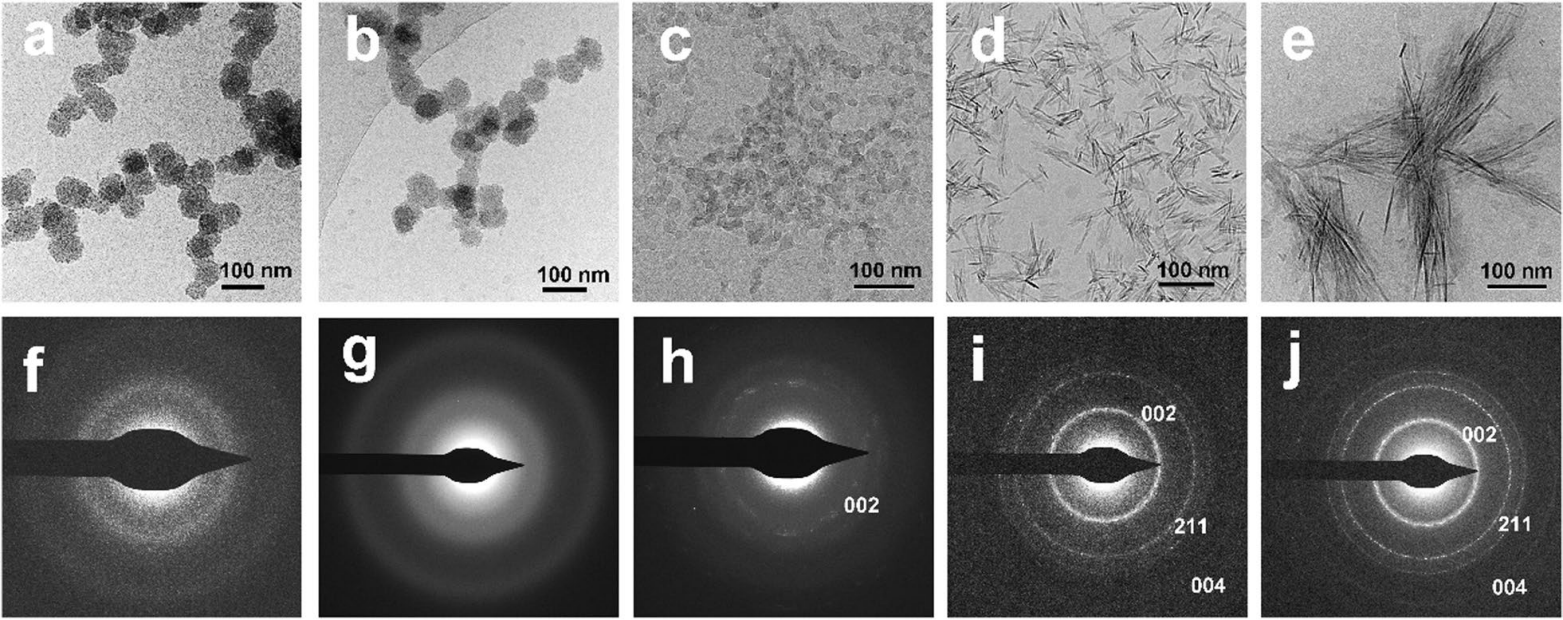
Cryo-TEM images of the mineralizing film and its SAED pattern at 0 h (**a**, **f**), 6 h (**b**, **g**), 8 h (**c**, **h**), 12 h (**d**, **i**) and 24 h (**e**, **j**) in artificial saliva. **a**, **b** The spherical ACP nanoparticles in the mineralizing film are approximately 30–80 nm in diameter and stable in artificial saliva over 6 h. **c** The ACP nanoparticles decreased in size and fused at 8 h. **d**, **e** Most of the ACP nanoparticles transformed into HAp at 12 and 24 h. **f**–**j** SAED patterns of the mineralizing film

Cryo-TEM images indicate that spherical ACP nanoparticles with diameters of approximately 30–80 nm remained amorphous within the first 6 h after the mineralizing film is incubated in artificial saliva (Fig. 4a, b, f, g). The nanoparticles decreased in size and adjacent ACP nanoparticles began to fuse; some of the nanoparticles are transformed into weakly crystallized HAp at 8 h (Fig. 4c, h). The reduced ACP nanoparticle size might have resulted from dehydration during crystallization processes. The ACP nanoparticles started to transform into needle-like HAp with continuous 002, 211, and 004 diffraction rings after 12 h incubation (Fig. 4d, i) and are mostly observed as HAp at 24 h (Fig. 4e, j).

Taken together, the ICP data, FTIR spectra, SF data and cryo-TEM images indicate that the mineralizing film not only provides calcium and phosphate sources but also transforms into HAp when exposed to artificial saliva at 37 °C over reasonable application times (6–8 h). These results imply that the mineralizing film could be conveniently used for dentin mineralization over 6–8 h during sleep [40].

### In vitro cytotoxicity tests of the mineralizing film

**Fig. 5:**
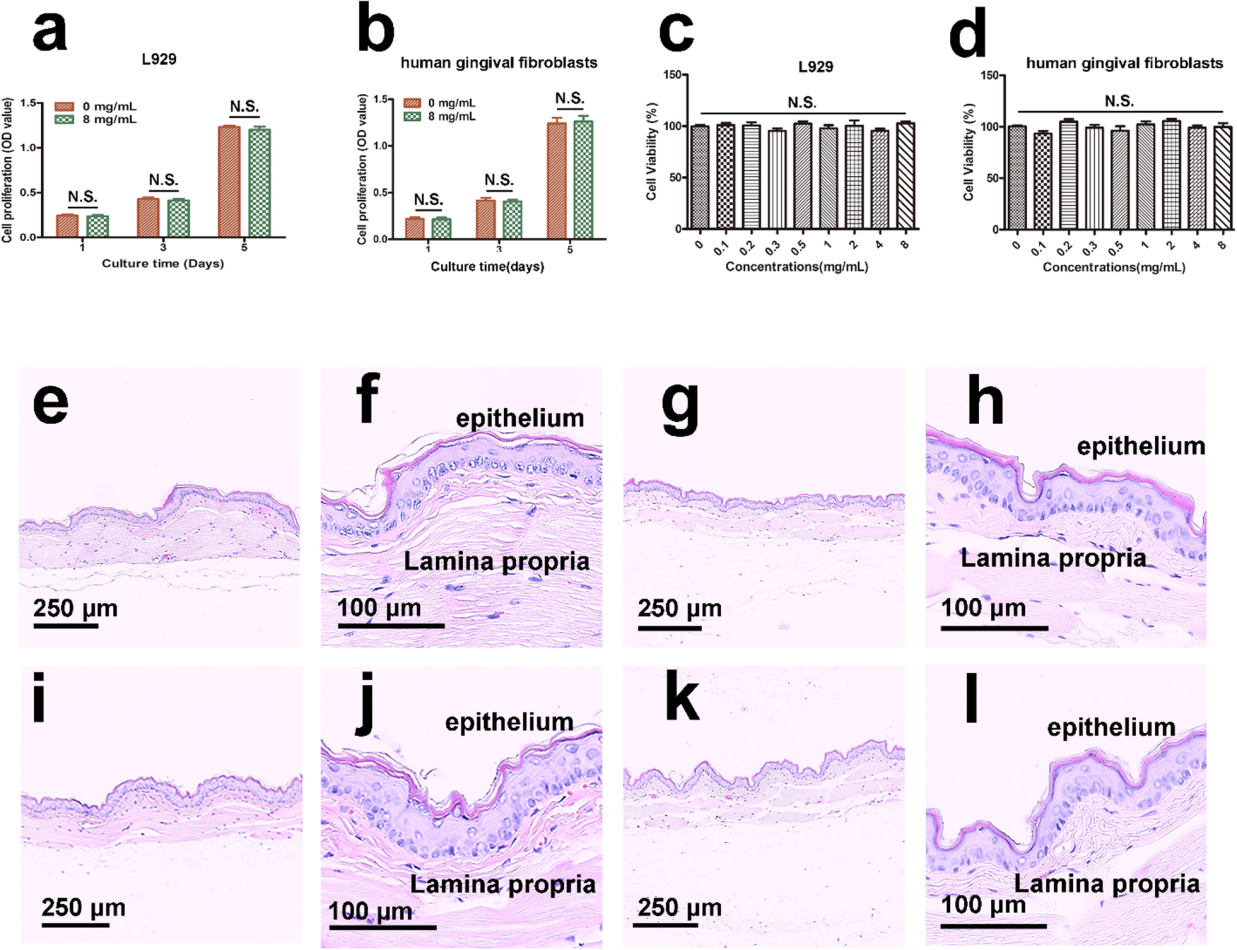
Cytotoxicity and oral mucosa irritation tests of the mineralizing film. **a**–**d** CCK-8 assay of L929 and human gingival fibroblasts. **e**–**l** Histological sections of oral mucosa from golden hamster cheek pouches. The oral mucosa was treated either with polar (0.9% NaCl) and nonpolar (cottonseed oil) liquid in the control group (**e**–**h**), or with the polar and nonpolar extracts of mineralizing film in the experimental group (**i**–**l**). **f**, **h**, **j**, and **l** are magnified images of **e**, **g**, **i**, and **k**. The stratified squamous epithelium and lamina propria were in normal arrangement. No cell proliferation, no edema, no inflammatory cells and no cell necrosis were detected

Cytotoxicity tests using L929 cells and human gingival fibroblasts, were performed with a CCK-8 assay (Fig. 5a–d). Compared with the control group, there were no significant differences in the proliferation of L929 cells and human gingival fibroblasts after 1, 3, and 5 days of incubation (Fig. 5a, b). After 3 days of incubation, both cell lines showed high cell viability rates in all different concentrations (Fig. 5c, d). The mineralizing film possesses excellent biocompatibility even at a high concentration (8 mg mL^−1^), which indicates that the mineralizing film could be used for biomedical applications. The results of the cytotoxicity tests performed in this study are consistent with the results of previous studies involving ACP nanoparticle-containing materials [41–45]

### Oral mucosa irritation tests of the mineralizing film

Mineralizing films should not irritate the oral mucosa if they are expected to be used in clinical applications [42]. The results of histological examinations are summarized in Additional file 1: Fig. S3, and representative histological images are shown in Fig. 5. The mean score was 0 for all the six groups (Additional file 1: Fig. S3). No visible proliferation, alteration, degeneration or necrosis of epithelial cells was observed. No pathological changes, such as congestion, edema, inflammatory infiltration or necrosis under the mucosa, were detected (Fig. 5e–l). Therefore, the mineralizing film does not irritate the mucosa [43, 46].

### In vitro experiments on the biomimetic mineralization of dentin

**Fig. 6:**
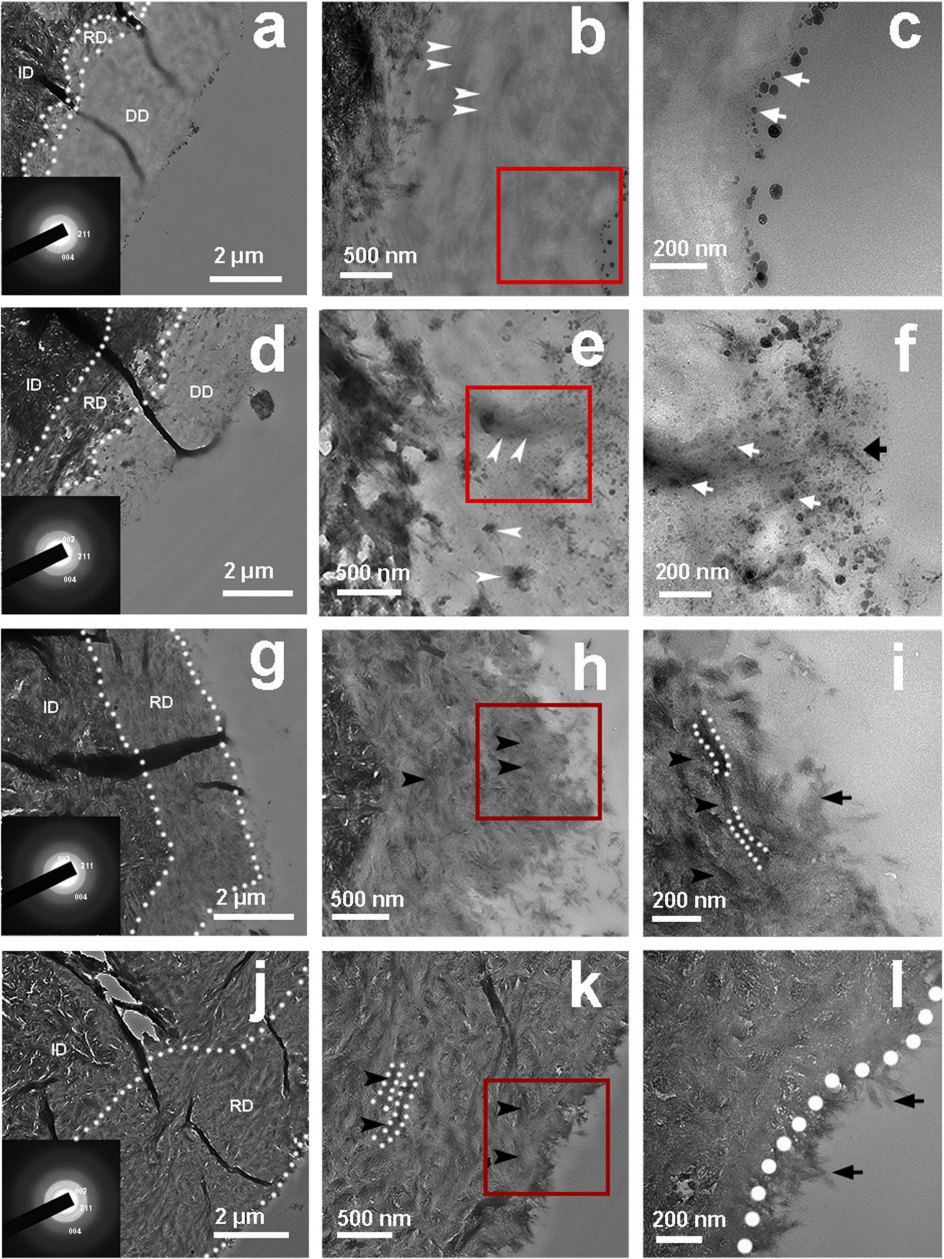
TEM images of dentin treated with the mineralizing film for 24 h (**a**–**c**), 48 h (**d**–**f**), 72 h (**g**–**i**) and 96 h (**j**–**l**). **c**, **f**, **i** and **l** are magnified images of **b**, **e**, **h** and **k**, respectively. At 24 h, spherical ACP nanoparticles were attached to the surface (**c**, white arrow). At 48 h, some nanoparticles (**f**, black arrow) were observed on the surface, and some were observed in the middle of the demineralized dentin layer (**e**, **f**, white arrow). The collagens became thicker and darker (**e**, white arrow). Rod-like crystals were detected on the surface of the remineralized dentin after 72 h (**i**, black arrow), and the demineralized dentin was fully mineralized and fused with the surface crystals of the dentin. A needle-like HAp layer was detected on the dentin surface at 96 h **(l**, black arrow). Both the black arrow and white dotted line (**k**, **l**) indicate the remineralized dentin collagen. ID, intact dentin; DD, demineralized dentin; RD, remineralized dentin

A mineralizing film was used as a carrier to achieve biomimetic mineralization of demineralized dentin. TEM images show that the remineralized dentin becomes darker and thicker over time. The SAED patterns (insets of Fig. 6a, d, g, j) reveal typical 002, 004 and 211 diffraction rings. A thin, weakly crystallized remineralization layer of approximately 0.3–0.5 µm was detected at the bottom of the demineralized dentin after 24 h incubation (Fig. 6a, white dotted line). The thickness of the remineralization layer increased to approximately 1–1.2-µm after 48 h incubation, and the layer exhibited regular crystallinity (Fig. 6d, white dotted line). After 72 h incubation, the thickness of the layer increased to approximately 2-µm, and the layer exhibited good crystallinity but a low electron density (Fig. 6g, white dotted line). Complete remineralization was observed after 96 h incubation, and the final thickness of the layer was that of the demineralized dentin (Fig. 6j, white dotted line). The final electron density of the remineralized dentin was similar to that of the neighboring, intact dentin. The SAED patterns show characteristic HAp planes, such as the 002, 211, and 004 planes (insets in Fig. 6a, d, g, j).

Furthermore, spherical ACP nanoparticles were attached to the surface of the demineralized dentin after 24 h of incubation (Fig. 6c, white arrow). After 48 h of incubation, some nanoparticles (Fig. 6f, black arrow) were present on the surface of the demineralized dentin, and some spherical ACP nanoparticles were observed in the middle of the demineralized dentin layer (Fig. 6e, f, white arrow). The collagen became increasingly thicker, inferring that some ACP nanoparticles penetrated the collagen (Fig. 6e, f white arrow). After 72 h of incubation, almost all of the nanoparticles detected on the surface of the remineralized dentin were rod-like in shape (Fig. 6i, black arrow). At 96 h, the demineralized dentin is fully remineralized and fused with the surface crystals of the dentin (Fig. 6l). A needle-like HAp layer was found attached to the dentin surface (Fig. 6l, black arrow). Therefore, it can be inferred that some of the ACP nanoparticles on the surface of the remineralized dentin transformed into a needle-like HAp layer with a thickness of 0.2 µm, and some of the ACP nanoparticles migrated through the demineralized layer and entered the collagen to promote intrafiber mineralization of the demineralized dentin.

**Fig. 7:**
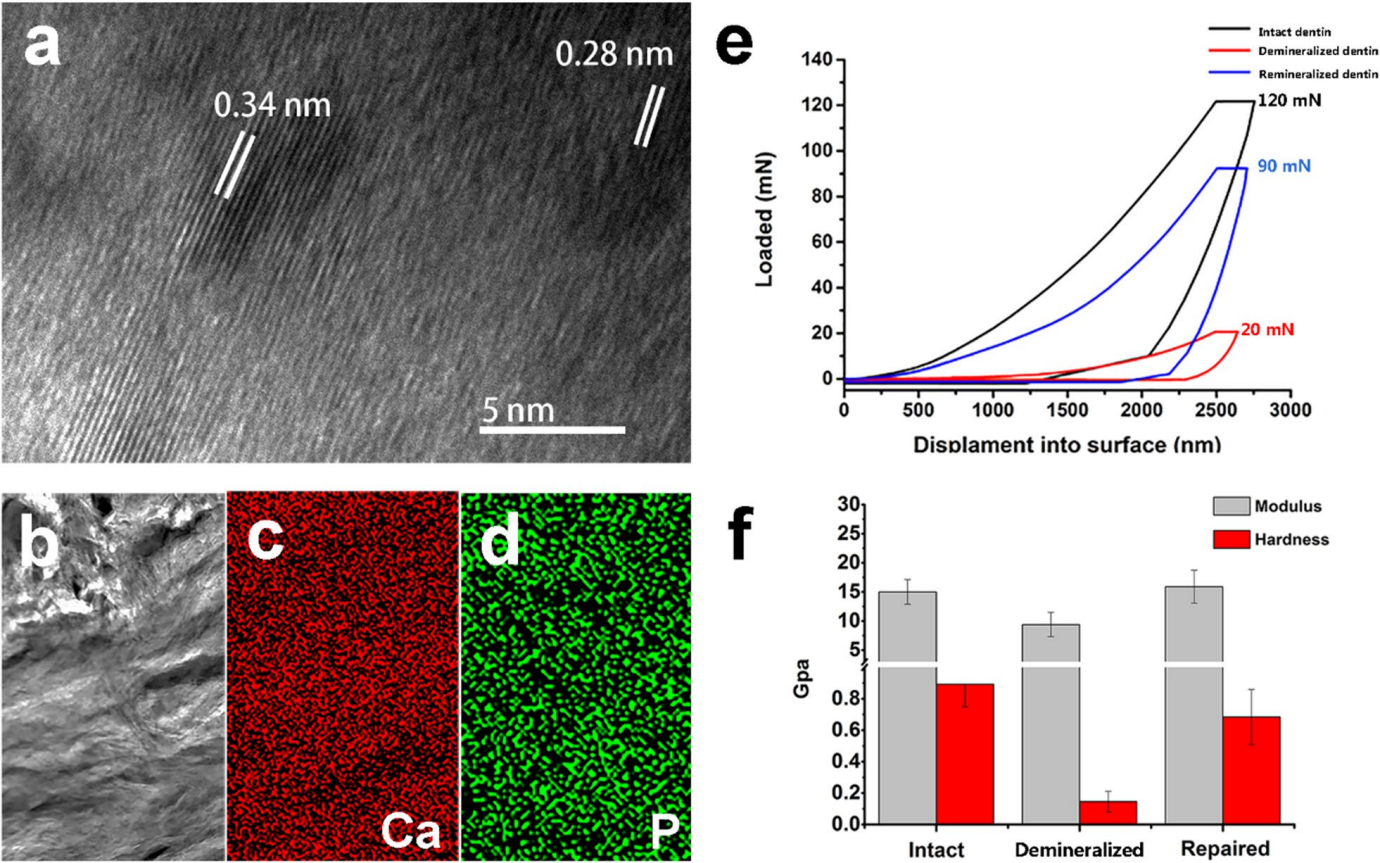
HRTEM images, elemental maps and nanoindentation tests of the dentin. **a** HRTEM image shows the two interplanar spacings: 0.34 nm and 0.28 nm. **b** Remineralized dentin. **c**, **d** Elemental maps revealing the uniform distribution of calcium and phosphate of **b**. **e** Load–displacement curves show the different load forces exerted on the intact dentin, demineralized dentin and remineralized dentin at the same dent depth (2500 nm). **f** Hardness and elastic modulus values for the intact dentin, demineralized dentin and remineralized dentin. Both the remineralized and intact dentin exhibited much higher values than the demineralized dentin

High-resolution transmission electron microscopy (HRTEM) images show two interplanar spacings: 0.34 nm and 0.28 nm; these spacings agree with those of the 002 and 211 HAp lattice planes of HAp, respectively (Fig. 7a). This is consistent with the findings of previous publications. Elemental maps also indicate that Ca and P are uniformly distributed in the remineralization layer (Fig. 7b–d). The different load forces exerted on the demineralized dentin (16 mN), the remineralized dentin (100 mN) and the intact dentin (120 mN) produced the same dent depth (2500 nm) (Fig. 7e). Furthermore, the hardness (0.68 ± 0.17 GPa) and elastic modulus (15.91 ± 2.84 GPa) of the remineralized dentin were nearly restored to those of the intact dentin (0.89 ± 0.14 GPa and 14.99 ± 2.12 GPa, respectively) (Fig. 7f). Both the remineralized and intact dentin exhibited much higher values than the demineralized dentin (0.14 ± 0.06 GPa and 9.42 ± 2.07GPa) (Fig. 7f).

In addition, mineralization of the demineralized dentin was not detected in the control groups even after 96 h incubation (Additional file 1: Figs. S4 and S5). These results might be attributed to the absence of ACP nanoparticles in the control groups. The HPMC film alone did not induce the mineralization of dentin.

### In vivo experiments on the biomimetic mineralization of dentin

**Fig. 8:**
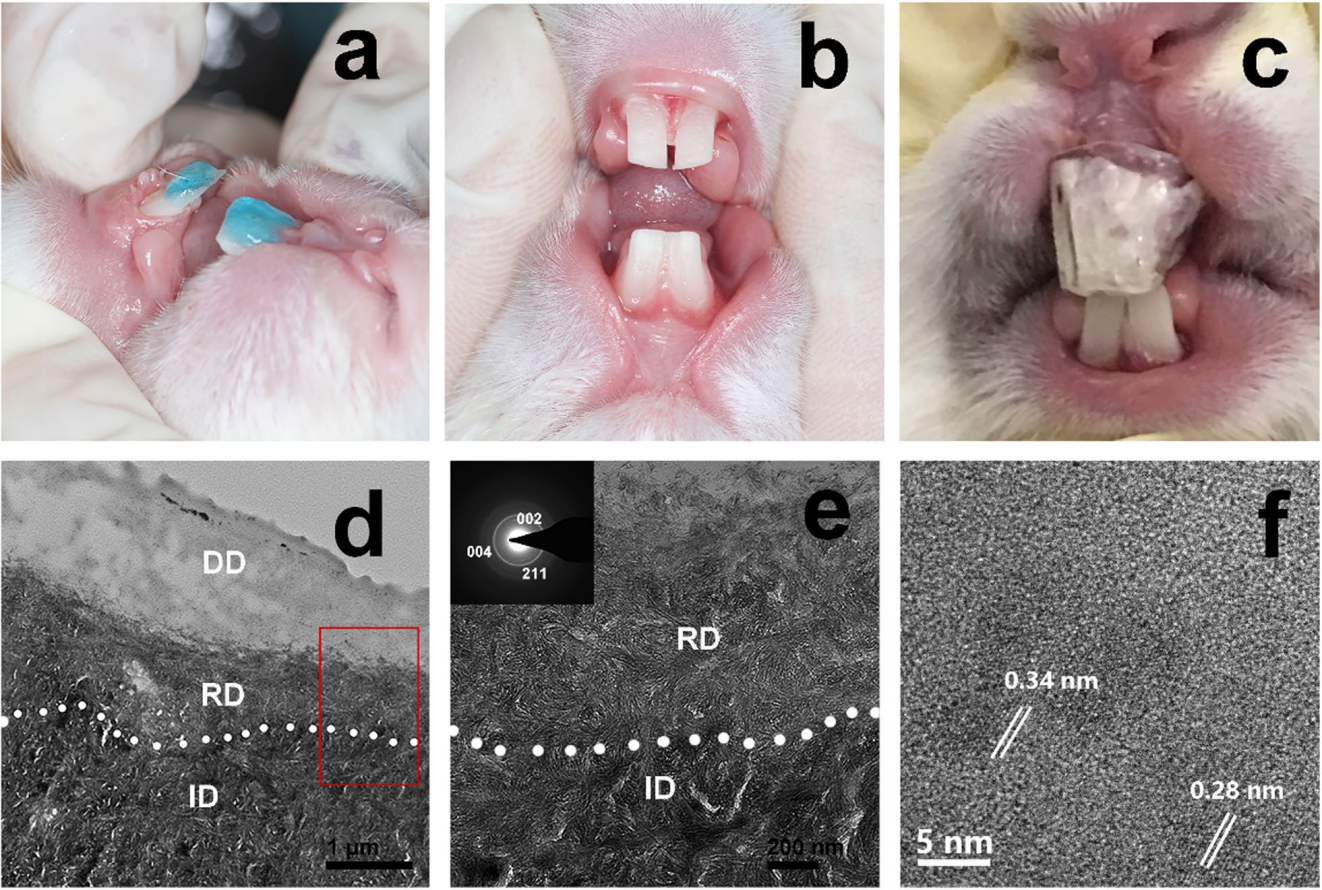
In vivo remineralization experiment of demineralized dentin of rabbits. **a** The rabbit dentins were etching by 37% phosphoric acid for 15 s. **b** The demineralized dentins were obtained after rinsing and gentle drying. **c** The mineralizing film attached on the demineralized dentin surface was covered with a transparent customized tray. **d**–**f** TEM images of the demineralized dentin treated with the mineralizing film for 7 days show that the remineralized layer was approximately 600 nm thick [**d**, the magnified images of **d** (**e**, **f**)]. The SAED patterns (insets in **e**) reveal 002, 004, and 211 diffraction rings. **f** The HRTEM image shows two interplanar spacings: 0.34 nm and 0.28 nm. ID, intact dentin; DD, demineralized dentin; RD, remineralized dentin

The mineralizing film was used in vivo to promote the remineralization of demineralized dentin in rabbits (Fig. 8a–c). This treatment induced a remineralization layer of approximately 0.6 µm at the bottom of the demineralized dentin after 7 days (Fig. 8d–f). However, no remineralization of the demineralized dentin occurred in the control group rabbits (treatment with the HPMC film alone) after 7 days (Additional file 1: Fig. S6). The results of the in vivo and in vitro studies are consistent.

### Discussion on the biomimetic mineralization of dentin

**Fig. 9:**
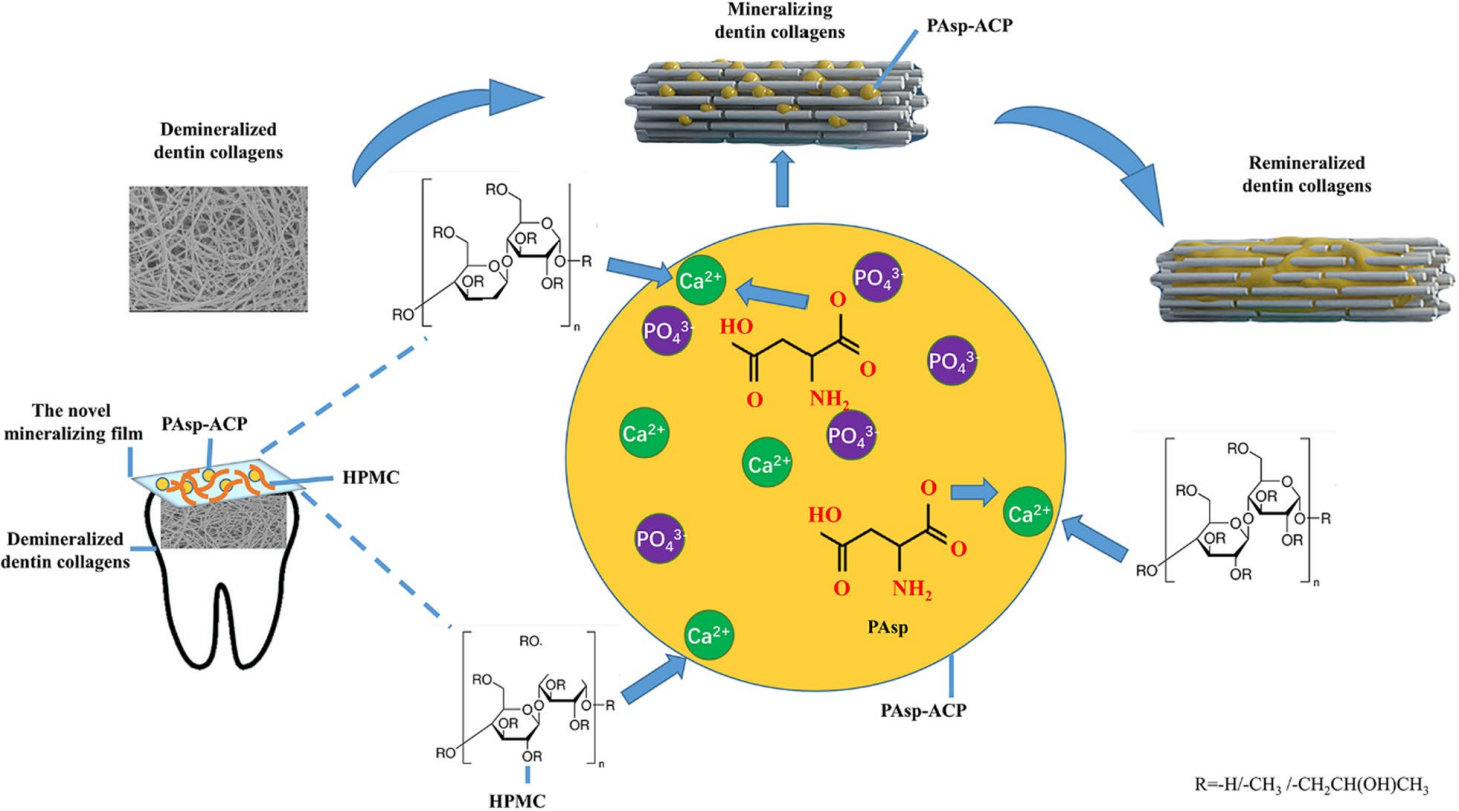
Diagram showing the mechanism of the remineralization of dentin collagens. Once in contact with water, the mineralizing film attaches to the demineralized dentin. The large number of hydroxyl, methyl and methoxy anion groups of the HPMC gel crosslink with calcium ions in synergy with PAsp. Finally, they stabilize the ACP precursors and induce the remineralization of dentin collagens

Unlike the biomimetic mineralization of bone, the cell processing is essential for the construction of the complex and hierarchical structures [47–51]. Biomimetic mineralization of dentin is mainly achieved by NCPs analogues via sequestering amorphous calcium phosphate precursors and inducing homogeneous apatite nucleation within the collagen fibrils [9]. As a major analog of NCPs, the carboxyl group of PAsp can combine with calcium ions to stabilize amorphous precursors and induce biomimetic mineralization [14, 15]. A large number of hydroxyl, methyl and methoxy anion groups of HPMC gel could also cross-link with calcium ions to form a stable network structure [23]. Liquid and paste used as carriers for current biomimetic mineralization could not achieve good clinical mineralization effectiveness due to lack of sustained release of Ca/P ions. In this study, HPMC loaded with PAsp-ACP nanoparticles could be dried to form a dry film, which is convenient for household use and carryon. HPMC plays an important role not only in stabilizing PAsp-ACP nanoparticles either in dry film or in gel status, but also in inducing the mineralization of dentin in gel status in synergism with PAsp additive (Fig. 9).

Once the HPMC film makes contact with water, it gradually becomes a gel due to its water adsorption [23]. Its polymer chains gradually relax and its volume simultaneously expands due to its interaction with hydroxyl groups of water [23, 24]. The broad band of the hydroxyl bond of HPMC (3460 cm^−1^) shifted to 3480 cm^−1^ of the HPMC-PAsp-ACP film (Additional file 1: Fig. S7). This behavior might be associated with the complexion between Ca^2+^ and polyhydroxyl groups of HPMC gel [23]. It is likely that compared with pure HPMC, the HPMC containing PAsp-ACP nanoparticles decreased the swelling ratio owing to the electrostatic attraction between Ca^2+^ ions and the polyhydroxyl of the HPMC (Additional file 1: Fig. S8). Furthermore, the granule spacer of the HPMC gel as interconnection control is critical issue for decreasing sedimentation process and sustaining release of ACP nanoparticles [52]. This is also supportive of extending phase transition time of PAsp-ACP nanoparticles in HPMC gel, compared with PAsp-ACP nanoparticles in AS (Additional file 1: Fig. S9). Furthermore, the hydrophobic microregions in HPMC gel promote the diffusion of the loaded nanoparticles by its hydrophobic methoxy groups [23, 24] They can provide a certain microenvironment for delivering PAsp-ACP precursors and controlling the diffusion of ions because the concentrations of Ca and phosphate ions increased in HMPC gel along with decreasing size of the of PAsp-ACP precursors (Fig. 4). The spherical PAsp-ACP nanoparticles along with calcium and phosphate ions penetrated into the demineralized layer due to concentration gradients (Fig. 6e, white arrow). They surrounded the demineralized collagen fibrils (Fig. 6e, f), gradually penetrated the collagen, and transformed into HAp. The process might be mediated by PAsp and HPMC. Finally, the demineralized dentin was heavily remineralized in vitro and in vivo (Figs. 6 and 8). The findings in this study might lay the foundation for a novel mineralization strategy in preventive dentistry.

## Conclusion

HPMC films can be used as novel amorphous precursor carriers to promote biomimetic mineralization. These mineralizing films which demonstrate excellent biocompatibility might pave the way for the design and fabrication of anti-carious materials for preventive dentistry.

## Supplementary Information

**Additional file 1: Section S1.** The preparation of demineralized dentin disks, dentin samples for TEM and nanoindentation test, the mineralizing film samples for cryo-TEM, The preparation of Cell isolation and culture of L929 and human gingival fibroblasts for CCK-8. The preparation of buccal mucosa samples for microscopic histological observations; The preparation of transparent custom trays; the swelling experiments. **Table S1.** Grading system for oral and penile reactions. **Table S2.** Grading system for microscopic examination for oral, penile, rectal and vaginal tissue reaction**. Figure S1** The photos of the mineralizing film in artificial saliva for 0, 1, 4, 6, 8, 10, 12 and 24 h at 37 °C. **Figure S2.** The splitting function (SF) of PAsp-ACP nanoparticles and the mineralizing film. **Figure S3.** The results of the histologic examination. **Figure S4.** TEM images of demineralized dentin (control group). **Figure S5.** TEM images of demineralized dentin treated with HPMC film (control group). **Figure S6.** TEM images of the demineralized dentin of rabbits treated with mineralizing film (control group). **Figure S7.** FTIR spectra of pure PAsp, PAsp-ACP, the pure HPMC film, the HPMC-PAsp-ACP film, a HPMC-CaCl_2_ film and a HPMC- Na_2_HPO_4_ film. **Figure S8** SEM images of HPMC gel and swelling ratios of HPMC and the mineralizing film. **Figure S9.** FTIR spectra of ACP in different systems.

## Data Availability

The datasets used and/or analyzed during the current study are available from the corresponding author upon reasonable request.
