# Correction to: Hydroxypropylmethylcellulose as a film and hydrogel carrier for ACP nanoprecursors to deliver biomimetic mineralization

**DOI:** 10.1186/s12951-021-01170-2

**Published:** 2021-12-18

**Authors:** Zhe Wang, Zihuai Zhou, Jiayan Fan, Leiqing Zhang, Zhixin Zhang, Zhifang Wu, Ying Shi, Haiyan Zheng, Zhengyi Zhang, Ruikang Tang, Baiping Fu

**Affiliations:** 1 Stomatology Hospital, School of Stomatology, Zhejiang University School of Medicine, Zhejiang Provincial Engineering Research Center for Oral Biomaterials and Devices, Zhejiang Provincial Clinical Research Center for Oral Diseases, Key Laboratory of Oral Biomedical Research of Zhejiang Province, Cancer Center of Zhejiang University, Hangzhou, 310006 China; 2 Department of Chemistry, Zhejiang University, Hangzhou, 310027 Zhejiang China

## Correction to: J Nanobiotechnol (2021) 19:385 https://doi.org/10.1186/s12951-021-01133-7

Following publication of the original article [1], the authors would like to correct the competing interest.

The correct version of competing interest is given in this erratum.

### **Competing interests**

Authors (B. Fu, Z. Wang, J. Fan and Z. Wu) declared that they have applied patents concerning mineralizing film for mineralization applications in China (No: ZL201610893384.3).

The original article is revised.
