# Supplementary material for: Hydroxypropylmethylcellulose as a film and hydrogel carrier for ACP nanoprecursors to deliver biomimetic mineralization
Source: J Nanobiotechnology. 2021 Nov 22;19:385. doi: 10.1186/s12951-021-01133-7 (PMC8607665; doi:10.1186/s12951-021-01133-7)
Supplement: Supplementary file 1 — Additional file 1: Section S1. The preparation of demineralized dentin disks, dentin samples for TEM and nanoindentation test, the mineralizing film samples for cryo-TEM, The preparation of Cell isolation and culture of L929 and human gingival fibroblasts for CCK-8. The preparation of buccal mucosa samples for microscopic histological observations; The preparation of transparent custom trays; the swelling experiments. Table S1. Grading system for oral and penile reactions. Table S2. Grading system for microscopic examination for oral, penile, rectal and vaginal tissue reaction. Figure S1 The photos of the mineralizing film in artificial saliva for 0, 1, 4, 6, 8, 10, 12 and 24 h at 37 °C. Figure S2. The splitting function (SF) of PAsp-ACP nanoparticles and the mineralizing film. Figure S3. The results of the histologic examination. Figure S4. TEM images of demineralized dentin (control group). Figure S5. TEM images of demineralized dentin treated with HPMC film (control group). Figure S6. TEM images of the demineralized dentin of rabbits treated with mineralizing film (control group). Figure S7. FTIR spectra of pure PAsp, PAsp-ACP, the pure HPMC film, the HPMC-PAsp-ACP film, a HPMC-CaCl2 film and a HPMC- Na2HPO4 film. Figure S8 SEM images of HPMC gel and swelling ratios of HPMC and the mineralizing film. Figure S9. FTIR spectra of ACP in different systems. [file 12951_2021_1133_MOESM1_ESM.docx]

**Additional file 1**

HydroxypropylMethylcellulose as a Film and Hydrogel Carrier for ACP Nanoprecursors to Deliver Biomimetic Mineralization

Zhe Wang,^1‡^ Zihuai Zhou,^1‡^ Jiayan Fan,^1^ Leiqing Zhang,^1^ Zhixin Zhang,^1^ Zhifang Wu,^1^ Ying Shi,^1^ Haiyan Zheng,^1^ Zhengyi Zhang,^1^ Ruikang Tang,^2^ and Baiping Fu,^1^*

1. Stomatology Hospital, School of Stomatology, Zhejiang University School of Medicine, Clinical Research Center for Oral Diseases of Zhejiang Province, Key Laboratory of Oral Biomedical Research of Zhejiang Province, Cancer Center of Zhejiang University, Hangzhou 310006
2. Department of Chemistry, Zhejiang University, Hangzhou, Zhejiang, 310027, China

**Additional Methods**

1. Preparation of demineralized dentin disks.^1^

2. Preparation of the dentin samples for TEM.^2^

3. Preparation of the mineralizing film samples for Cryo-TEM.^3^

4. Cell isolation and culture of L929 and human gingival fibroblasts for CCK-8.^4^

5. Preparation of dentin samples for nanoindentation test.^5^

6. Preparation of buccal mucosa samples for microscopic histological observations.^6^

7. Grading system for macroscopic and microscopic histological observations of Oral Mucosal Stimulation Test.^6^

8. Preparation of transparent custom trays.^7^

9. Swelling experiments.^8^

**1. Preparation of demineralized dentin samples**

- 1. **In vitro experiment**

Forty freshly-extracted human third molar teeth were collected with the patients’ informed consent. The protocol in this study was approved by our Institutional Ethics Committee. The coronal disks of the third molar teeth were prepared as 5mm×5mm×1mm with a slow-speed Isomet saw under copious cooling water. After the dentin surfaces were polished with 600-grit silicon carbide paper under running water, Thirty-six dentin disks were etched with 37% phosphoric acid for 10 s to completely demineralized, another four intact dentin disks were for nanoindentation testing.

- 1. **In vivo experiment**

The labial enamel surfaces of the six rabbits’ maxillary and mandibular central incisors were removed to expose the dentin，and the exposed dentin surfaces were etched with 37% phosphoric acid gel for 15 s and water rinsed thoroughly.

**2. Preparation of the dentin samples for TEM**

The remineralized dentin samples were fixed in 2.5% glutaraldehyde overnight before they were each rinsed with 2 mL of phosphate buffer saline (PBS). The samples were dehydrated in a gradient series of ethanol (30%, 50%, 70%, 90%), 15 min each, absolute ethanol and acetone 20 min each, before they were embedded in epoxy resin and kept at 70°C in an oven overnight. Ultrathin sections (90~110 nm) of dentin samples were prepared and analyzed by TEM (JEM-1230, JEOL, Tokyo, Japan) with selected area electron diffraction (SAED), high resolution transmission electron microscopy (HRTEM, JEM-2100F, JEOL, Tokyo, Japan).

**3. Preparation of the mineralizing film samples for Cryo-TEM**

0.4 g of mineralizing film was stored in 8 mL of artificial saliva at 37°C. After 2.5 μL of the supernatant was retrieved at 0, 6, 8,12 and 24 hrs, it was dripped to a holey carbon grid, blot-dried with filter paper and instantly plunged into liquid ethane and liquid nitrogen temperature in order to quickly freeze the samples and not to damage molecular features. After specimen is frozen, samples must be submerged in liquefied ethane and nitrogen.

**4. Cell isolation and culture of L929 and human gingival fibroblasts for CCK-8**

L929 were cultured with Dulbecco’s modified Eagle’s medium(DMEM) supplemented with 10% fetal calf serum (FCS). Human gingival tissue was obtained from a healthy patient according to the Medical Ethics Committee of Stomatology Hospital, Zhejiang University School of Medicine (file number 201905). The specimen was washed carefully three times with phosphate-buffered saline (PBS). After the washing process, the sample was placed in a cell culture dish with DMEM with 15% FCS supplemented with 50 μg/ml gentamycin and 10mML-glutamine. After 10 days, outgrown fibroblasts (GF-1) were harvested.

**5. Preparation of dentin samples for nanoindentation test**

Natural dentin, demineralized dentin disks (n=4) were prepared as abovementioned, the remineralized dentin disks of 4 d (n=4) were slightly polished by 2000 grit silicon carbide paper for 1s to get a smooth surface. Each sample with the polished surface upwards fixed on the nanoindentation bench.

**6. Preparation of buccal mucosa samples for microscopic histological observations.**

All experimental procedures are performed in accordance with the International Ethical Guidelines of the Declaration of Helsinki (World Medical Association, 2008) and National Ethics Censorship of Biomedical Research Involving Human Subject and approved by Ethical Committee of Zhejiang University. (No. 16113). Golden hamsters were sacrificed after the aforementioned treatments, the entire cheek pouch tissue were removed and fixed flat on a board by pinning the mucous membrane upward, and the sarcolemma surface downward. Afterward, the tissues were fixed in 10% (V/V) formalin, and stained by eosin-hematoxylin for pathological analysis. Three tissue sections of each sample were observed.

**7. Grading system for macro- and micro-scopic histological observations**

According to “Stimulation and Skin Allergy Test, Appendix B.3 Oral Mucosal Stimulation Test (ISO 10993-10, 2010)”, First, we make some macroscopic observations —described the appearance of the cheek pouches for each animal, decided the pouch surface erythema grades at each time interval according to Table S1. Finally, the microscopically irritant effects on oral tissue would be evaluated by a pathologist, and grade each sample according Table S2.

| Reaction | Numerical gradinng |
| --- | --- |
| Erythema and formation |  |
| No erythema | 0 |
| Very slight erythema (barely perceptible) | 1 |
| Well-defined erythema | 2 |
| Moderate erythema | 3 |
| Severe erythema (beet-redness) to eschar formation | 4 |
| Other adverse changes of the tissures should be recorded and reported |  |

**Table S1** Grading system for oral and penile reactions

| Reaction | Degree | Numerical grading |
| --- | --- | --- |
| Epithelium | Normal, intact  Cell degeneration or flatting  Metaplasia  Focal erosion  Generalized erosion | 0  1  2  3  4 |
| Leucocyte infiltration (per high power field) | Absent  Minimal (less than 25)  Mild (26 to 50)  Moderate (51 to 100)  Marker (greater than 100) | 0  1  2  3  4 |
| Vascular congestion | Absent  Minimal  Mild  Moderate  Marked with disruption of vessels | 0  1  2  3  4 |
| Oedema | Absent  Minimal  Mild  Moderate  Marked | 0  1  2  3  4 |

**Table S2** Grading system for microscopic examination for oral, penile, rectal and vaginal tissue reaction

**8. Preparation of transparent custom trays**

The impression of the Rabbits’ maxillary central incisor was taken by silicone rubber impression materials and stone casts were prepared. Afterward, the transparent custom tray was made with the polymer transparent vacuum suction membranes under the vacuum laminsator.

**9. Swelling experiments**

Swelling behavior of HPMC/HPMC-PAsp-ACP hydrogels was determined by immersing the dried hydrogels in distilled water at room temperature and measuring their weight at different time intervals (2h, 4h, 6h). The swollen samples were removed from water and weighed periodically after the excess water was wiped offusing absorbent paper. The swelling ratio at different times, can be calculated as follows (where W_t_ and W_0_ represent the weight of the prepared hydrogels in wet and dry state, respectively), Swelling ratio (%) = 𝑊_t_ – 𝑊_0_/ 𝑊_0_ ×100%.

**Additional Figures**

**
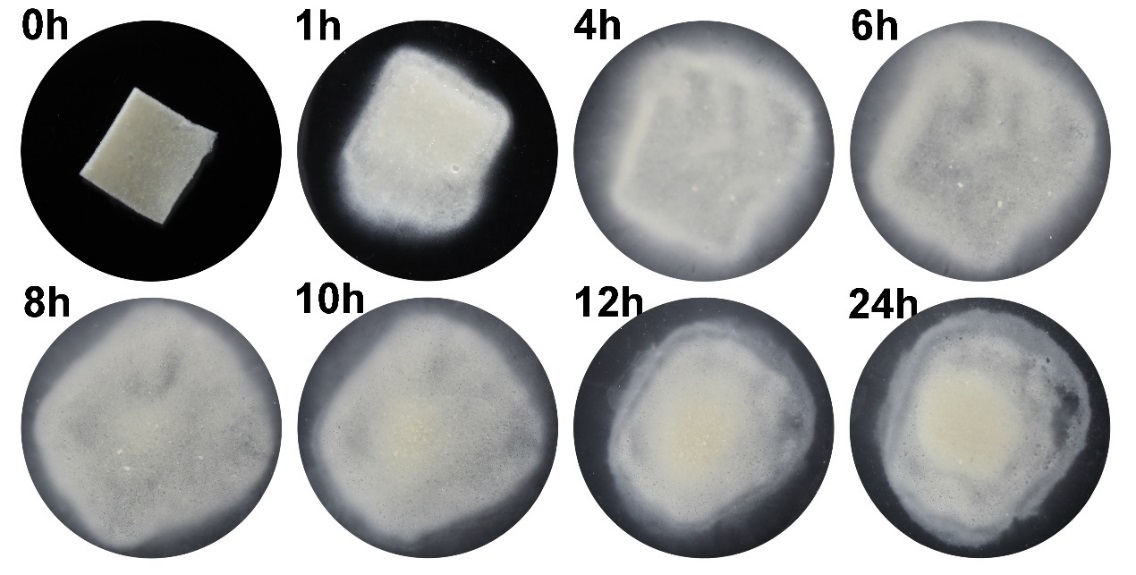
**

**Fig. S1.** The photos of the mineralizing film in artificial saliva for 0, 1, 4, 6, 8, 10, 12 and 24 hrs at 37°C, revealing that the status of mineralizing film dynamically changed from the dry film to gel at 4 h and its macro-morphological changes over 6 to 24 h.


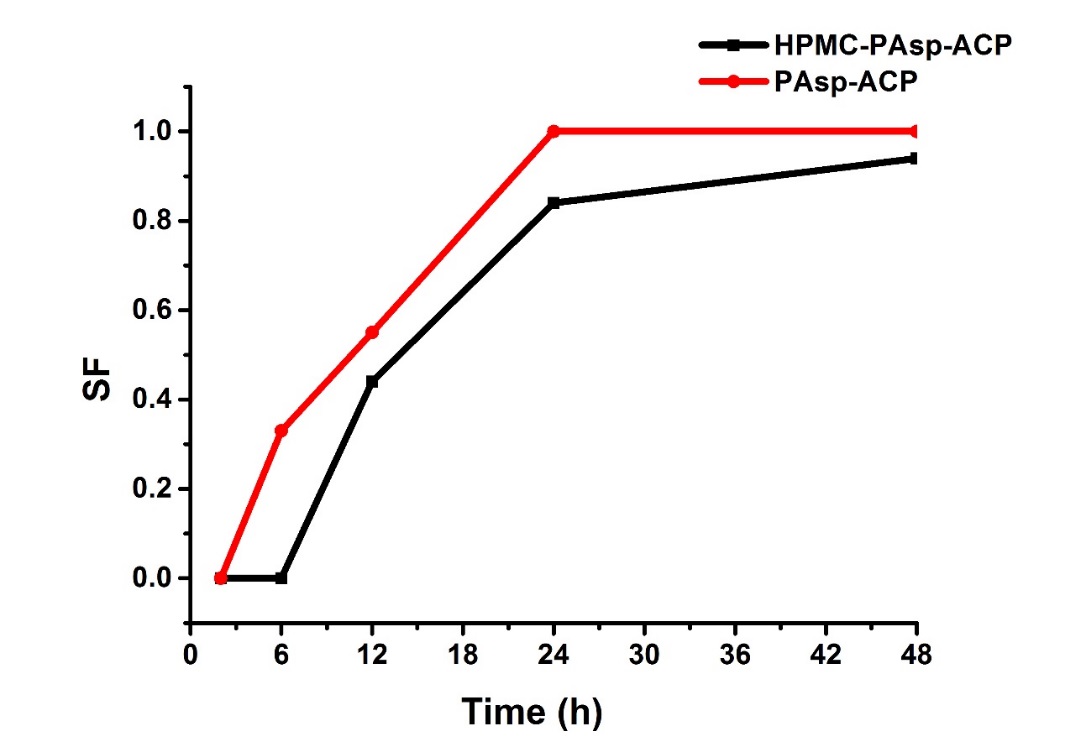


**Fig. S2.** the splitting function (SF) of PAsp-ACP nanoparticles (red line) and the mineralizing film (HPMC loaded PAsp-ACP nanoparticles) (black line) for 2, 6, 12, 24, 48h in artificial saliva at 37°C.

| Animal number | Cheek pouch | Epithelial change | Leukocyte infiltration | Blood vessel congestion | Edema | total points |
| --- | --- | --- | --- | --- | --- | --- |
| 101 | Right  0.9% NaCL (control) | 0 | 0 | 0 | 0 | 0 |
|  | Left experiment | 0 | 0 | 0 | 0 | 0 |
| 102 | Right  0.9% NaCL (control) | 0 | 0 | 0 | 0 | 0 |
|  | Left experiment | 0 | 0 | 0 | 0 | 0 |
| 103 | Right  0.9% NaCL (control) | 0 | 0 | 0 | 0 | 0 |
|  | Left experiment | 0 | 0 | 0 | 0 | 0 |
| means | Right  0.9% NaCL (control) | 0 | | | | |
|  | Left experiment | 0 | | | | |
| 104 | Right  cottonseed oil (control) | 0 | 0 | 0 | 0 | 0 |
|  | Left experiment | 0 | 0 | 0 | 0 | 0 |
| 105 | Right  cottonseed oil (control) | 0 | 0 | 0 | 0 | 0 |
|  | Left experiment | 0 | 0 | 0 | 0 | 0 |
| 106 | Right  cottonseed oil (control) | 0 | 0 | 0 | 0 | 0 |
|  | Left experiment | 0 | 0 | 0 | 0 | 0 |
| means | Right  cottonseed oil (control) | 0 | | | | |
|  | Left experiment | 0 | | | | |

**Fig. S3.** The results of the histologic examination

**
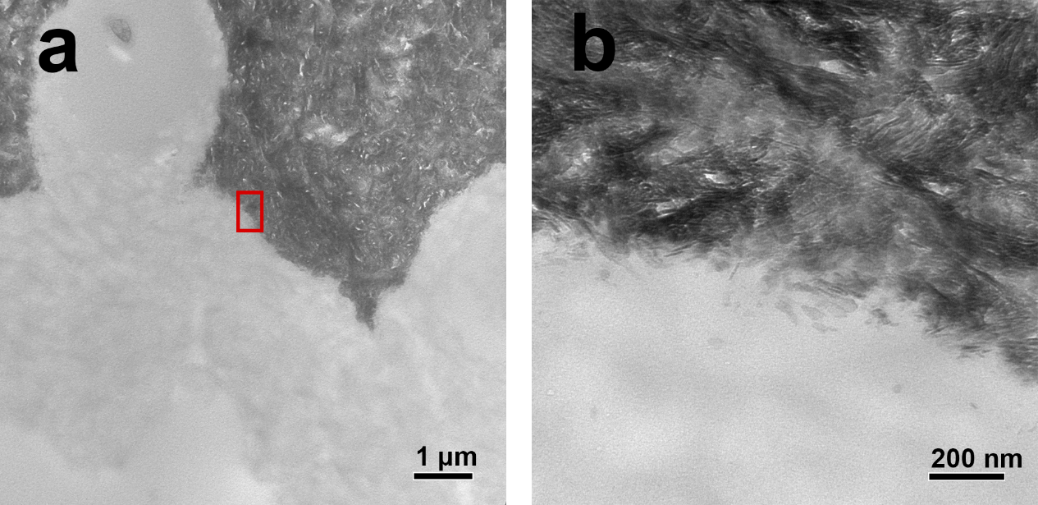
**

**Fig. S4.** TEM images of demineralized dentin in artificial saliva for 96 hrs. Panels b are the higher magnifications of the red box areas in panels a. No obvious mineralization was detectable.

**
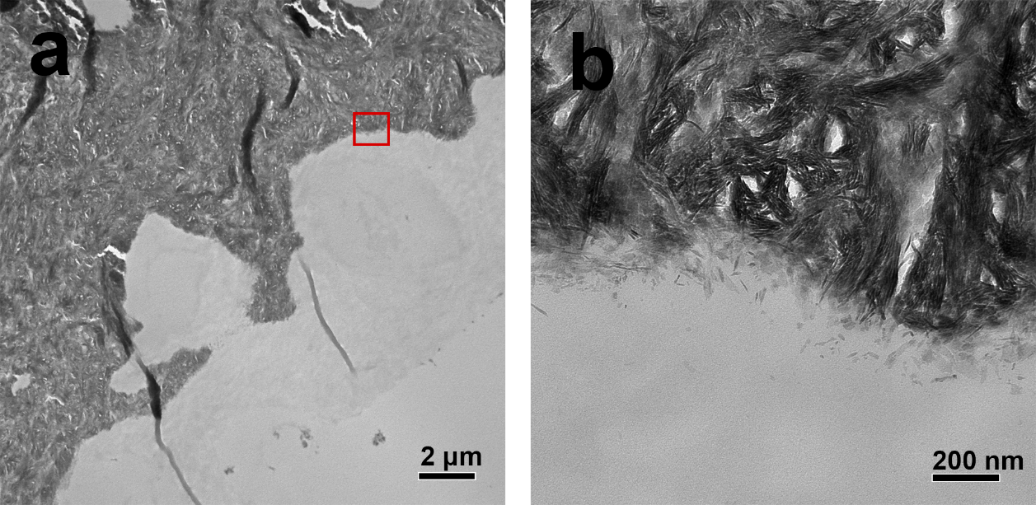
**

**Fig. S5.** TEM images of demineralized dentin treated with HPMC film in artificial saliva for 96 hrs. Panels b are the higher magnifications of the red box areas in panels a. No obvious mineralization was detectable.

**
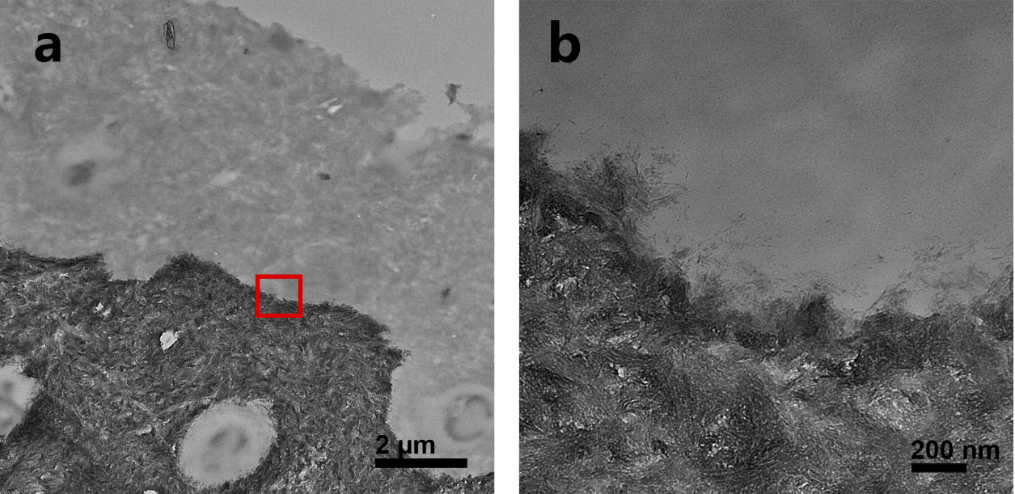
**

**Fig. S6.** TEM images of the demineralized dentin of rabbits treated with mineralizing film for 7 d (control group). Panels b are the higher magnifications of the red box areas in panels a. No obvious mineralization was detectable.


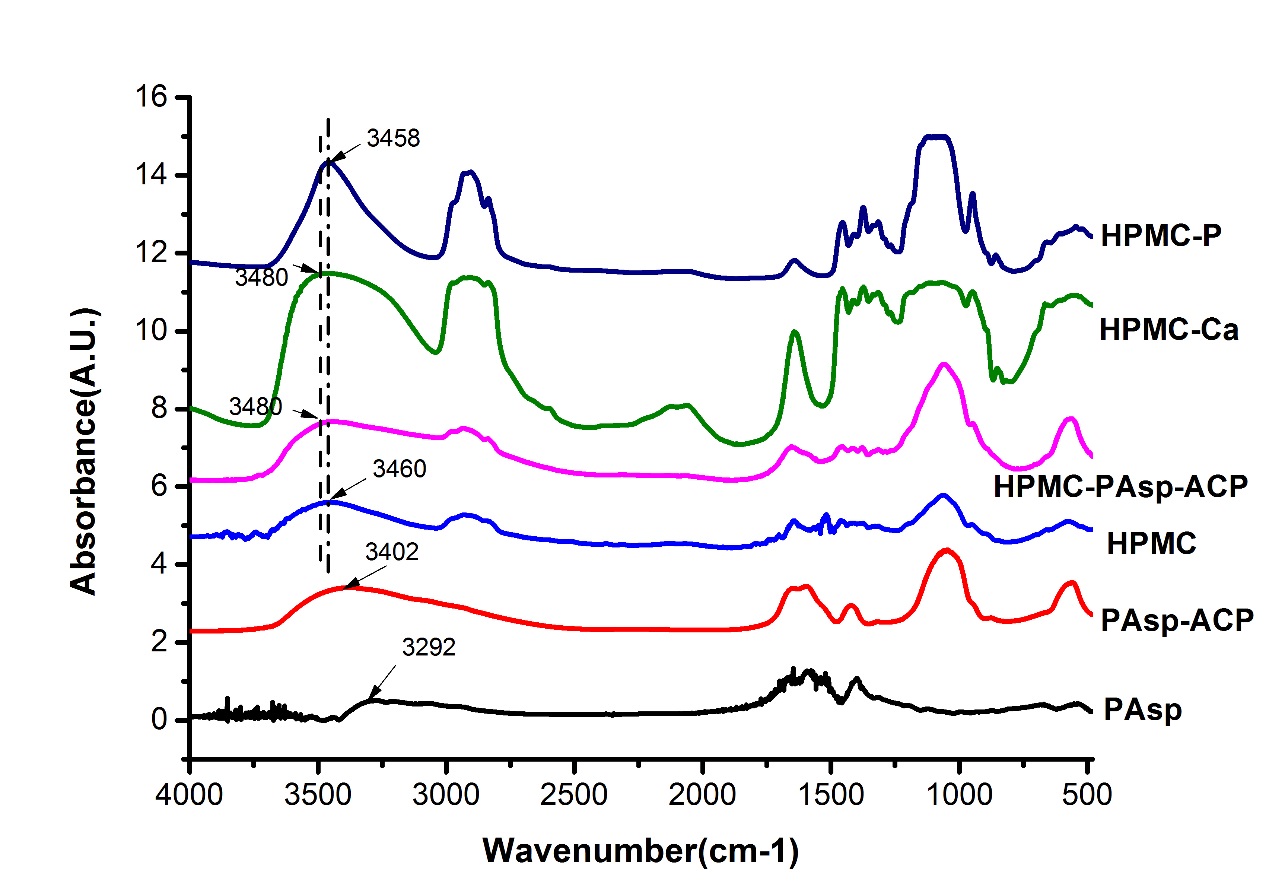


**Fig. S7.** FTIR spectra of pure PAsp, PAsp-ACP, the pure HPMC film, the HPMC-PAsp-ACP film, a HPMC-CaCl2 film and a HPMC- Na_2_HPO_4_ film. The absorption band corresponding to the hydroxyl of the carboxyl group of pure PAsp (3292 cm^-1^) evidently shifted to 3402 cm^-1^ in PAsp-ACP. The broad band corresponding to hydrogen bonding in HPMC (3460 cm^-1^) shifted to 3480 cm^-1^ in the HPMC-PAsp-ACP film and the HPMC-CaCl_2_ film. The band corresponding to hydrogen bonding in the HPMC Na_2_HPO_4_ film (3458 cm^-1^) was consistent with that of pure HPMC.


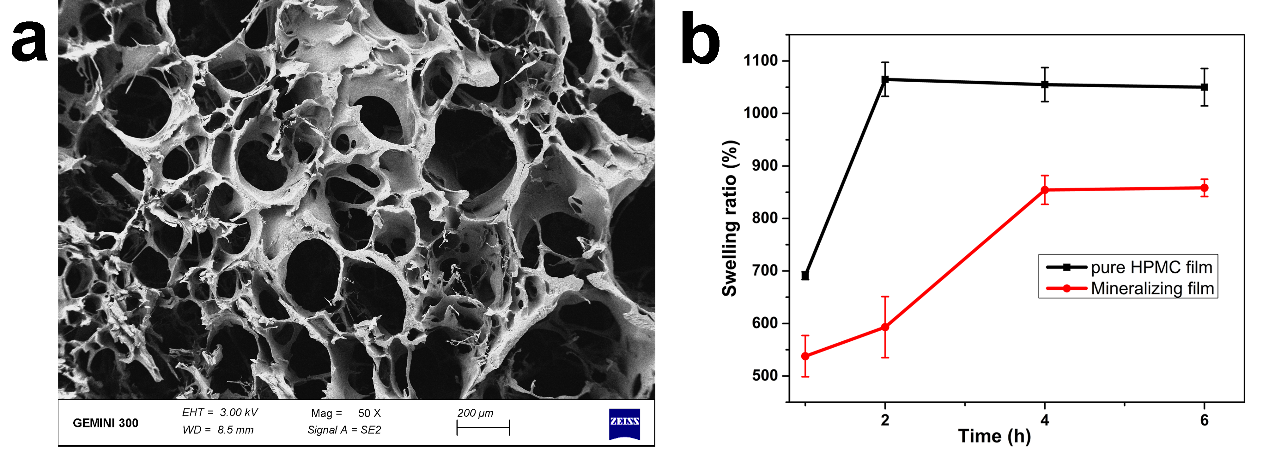


**Fig. S8** The SEM image of pure HPMC gel and swelling ratio of HPMC and the mineralizing film (HPMC-PAsp-ACP). SEM shows the porous microstructure of HPMC gel. Pure HPMC film swells completely after 2 hours, the mineralizing film swells completely after 4 hours.


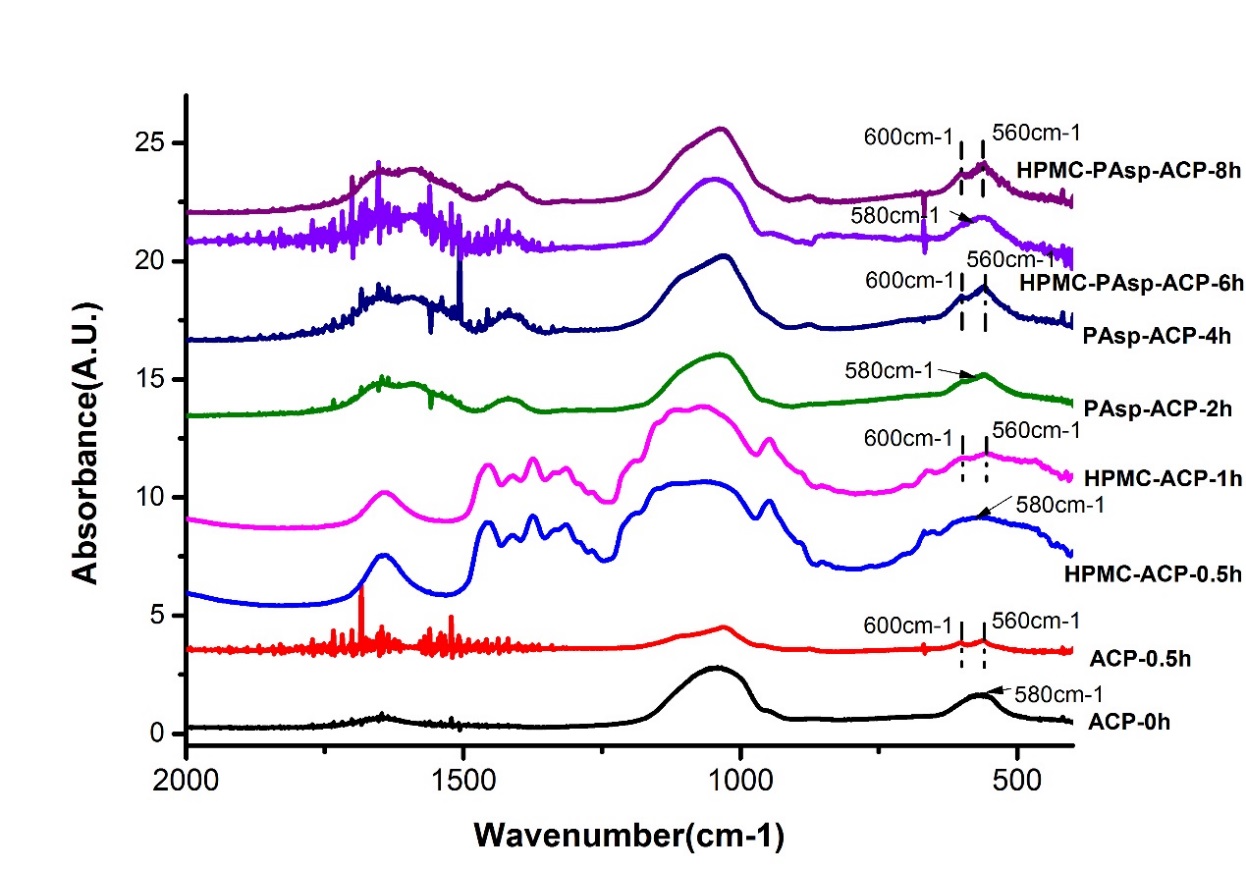


**Fig. S9.** FTIR spectra of ACP in different systems. The ACP precursors were unstable and transformed into HAp in artificial saliva within 30 min. When ACP was dispersed in the HPMC matrix (in the absence of PAsp), the phase transition in artificial saliva was prolonged to between 30 min and 1 hr. However, ACP could be effectively stabilized by PAsp for over 2 hrs. Furthermore, the ACP nanoparticles remained amorphous for over 6 hrs in the PAsp-ACP-HPMC film.

**Addditional References**

1. Van Meerbeek B, Conn LJ Jr, Duke ES, Eick JD, Robinson SJ, Guerrero D. Correlative transmission electron microscopy examination of nondemineralized and demineralized resin-dentin interfaces formed by two dentin adhesive systems. J Dent Res. 1996; 75(3) :879-88.

2. Osipov VY, Enoki T, Takai K, Takahara K, Endo M, Hayashi T, et al. Magnetic and high resolution TEM studies of nanographite derived from nanodiamond. Carbon. 2006; 44(7): 1225-1234.

3. Cheng Y, Grigorieff N, Penczek PA, Walz T. A primer to single-particle cryo-electron microscopy. Cell. 2015; 161(3): 438-449.

4.  Schubert A, Ziegler C, Bernhard A, Bürgers R, Miosge N. Cytotoxic effects to mouse and human gingival fibroblasts of a nanohybrid ormocer versus dimethacrylate-based composites. Clin Oral Investig. 2019; 23(1): 133-139.

5. Meredith N, Sherriff M, Setchell DJ, Swanson SA. Measurement of the microhardness and Young's modulus of human enamel and dentine using an indentation technique. Arch Oral Biol 1996; 41:539-45.

6. Institution BS. Biological evaluation of medical devices. Tests for irritation and skin sensitization. 1994.

7. Han M, Li QL, Cao Y, Fang H, Xia R, Zhang ZH. In vivo remineralization of dentin using an agarose hydrogel biomimetic mineralization system. Sci Rep. 2017; 7: 41955.

8. Feng X., Hou, X., Cui C., Sun S., Sadik, S, Wu S and Zhou F. Mechanical and antibacterial properties of tannic acid-encapsulated carboxymethyl chitosan/polyvinyl alcohol hydrogels. Eng Regener. 2021; 2: 57-62.
